# Absence of N-terminal acetyltransferase diversification during evolution of eukaryotic organisms

**DOI:** 10.1038/srep21304

**Published:** 2016-02-10

**Authors:** Om Singh Rathore, Alexandra Faustino, Pedro Prudêncio, Petra Van Damme, Cymon J. Cox, Rui Gonçalo Martinho

**Affiliations:** 1 Department of Biomedical Sciences and Medicine, Faro, Portugal; 2 Center for Biomedical Research (CBMR), Faro, Portugal; 3 ProRegeM-PhD Program in Mechanisms of Disease and Regenerative Medicine, Faro, Portugal; 4 Center of Marine Sciences, University of Algarve, Faro, Portugal; 5 Instituto Gulbenkian de Ciência, Rua da Quinta Grande 6, Oeiras 2781-901, Portugal; 6 Department of Medical Protein Research, VIB, B-9000 Ghent, Belgium; 7 Department of Biochemistry, Ghent University, B-9000 Ghent, Belgium

## Abstract

Protein N-terminal acetylation is an ancient and ubiquitous co-translational modification catalyzed by a highly conserved family of N-terminal acetyltransferases (NATs). Prokaryotes have at least 3 NATs, whereas humans have six distinct but highly conserved NATs, suggesting an increase in regulatory complexity of this modification during eukaryotic evolution. Despite this, and against our initial expectations, we determined that NAT diversification did not occur in the eukaryotes, as all six major human NATs were most likely present in the Last Eukaryotic Common Ancestor (LECA). Furthermore, we also observed that some NATs were actually secondarily lost during evolution of major eukaryotic lineages; therefore, the increased complexity of the higher eukaryotic proteome occurred without a concomitant diversification of NAT complexes.

The genetic code is almost universal and the decoding molecular machine, the ribosome, is highly conserved across all known living organisms^1^. The narrow dimension of the ribosome exit tunnel (the cavity from which the nascent peptide emerges) precludes large domain folding of the nascent protein. This creates a window of opportunity for modification of protein residues that would be otherwise inaccessible due to folding. Indeed, co-translational modifications are widespread in cells throughout all three traditional kingdoms of life. Among others, these modifications include the proteolytic excision of the initial methionine and protein N-terminal acetylation (Nt-acetylation)^2^,^3^,^4^, which involves the transfer of an acetyl group from acetyl-CoA to the protein alpha-amino group^4^. Although Nt-acetylation is an ubiquitous modification in eukaryotes, its prevalence varies, having a protein frequency of 50–70% in *Saccharomyces cerevisiae* (budding yeast), 70–80% in *Drosophila melanogaster* (fruit fly), and 80–90% in *Homo sapiens* (humans) and *Arabidopsis thaliana* (flowering plant)^5^,^6^,^7^,^8^,^9^. In eubacteria typically less than 10% of proteins are (partially) N-terminally acetylated, whereas in archaeal species it varies between 14–29% of all studied proteins^3^,^10^.

Nt-acetylation may influence protein half-life^11^,^12^,^13^,^14^,^15^, localization and export^16^,^17^, protein-protein and protein-lipid interactions^18^,^19^,^20^,^21^,^22^, the correct organization and function of the cellular cytoskeleton^23^,^24^,^25^, nuclear chromatin^26^,^27^, and vesicular compartment^28^. Its mis-regulation is frequently associated with tumor development and aggressiveness^29^, and distinct human syndromes^30^,^31^,^32^,^33^.

Nt-acetylation is catalyzed by a highly conserved family of N-terminal acetyltransferases (NATs). Prokaryotes have at least three NATs^3^,^34^,^35^,^36^,^37^, whereas *H. sapiens* has six distinct but highly conserved NATs (NatA-F) (Fig. 1A). While some of these NATs are protein complexes requiring different catalytic and auxiliary subunits (e.g. NatA, NatB, and NatC), other NATs are able to Nt-acetylate independently of protein partners (e.g. NatD, NatF, and possibly NatE)^9^,^25^,^38^,^39^,^40^,^41^,^42^,^43^,^44^,^45^,^46^,^47^. NATs have distinct substrate specificity profiles, where substrate recognition depends on the identity of the first 2–5 amino acids of the elongating polypeptide^6^,^9^,^39^,^48^,^49^,^50^,^51^.

Nascent proteins are synthesized with a N-terminal methionine (also known as the initiator methionine or iMet), but if the second residue is non-bulky the iMet is frequently co-translationally removed by methionine aminopeptidases and the second residue is Nt-acetylated by NatA^6^,^49^,^50^. If the iMet is not excised, it can be Nt-acetylated by the other NATs. NatA and NatB are the major NATs in eukaryotic cells, which together Nt-acetylate approximately 60% of all *H. sapiens* proteins, while NatC, NatE, and NatF together Nt-acetylate only 15–20% of the proteome^9^,^52^. By contrast, the single archaeal NAT, possibly a direct ancestor of the eukaryotic NATs, has the ability to Nt-acetylate both NatA and NatE-type substrates of eukaryotes^34^. Such ancestral relationship implies the evolution of NAT substrate specialization and diversification in the eukaryote lineage.

Although absent in *S. cerevisiae*, NatF has been identified in *H. sapiens*, *D. melanogaster*, and more recently in *A. thaliana*^9^,^53^. NatF enzymatic activity is responsible for a significant increase in proteome Nt-acetylation when comparing *S. cerevisiae* to *H. sapiens*^9^, and specifically targets transmembrane proteins^28^. Indeed, since the number and degree of Nt-acetylated proteins and NAT diversity is higher in *H. sapiens* and *D. melanogaster* compared to *S. cerevisiae* and prokaryotes, it has been proposed that an increase in the regulatory complexity of this co-translational modification has occurred during evolution of higher eukaryotes^9^,^28^. Yet, the precise nature of these changes and their functional consequences remains poorly understood, as genome-wide studies across the eukaryotic tree of life are lacking.

In this work, we investigated the diversification of NATs during evolution of eukaryotic organisms. We concluded that most diversification of NATs happened before the evolution of eukaryotes, as our data strongly suggest that all six major human NATs were most likely present in the Last Eukaryotic Common Ancestor (LECA). Furthermore, we also observed that some NATs were secondarily lost during evolution of major eukaryotic lineages. Therefore, and although some clade-specific NAT duplications exist across the eukaryotic tree of life^54^,^55^,^56^, the increased complexity of the higher eukaryotic proteome occurred without a concomitant diversification of the major NAT complexes.

## Results

### All six major human NATs (NatA-F) were most likely present in the Last Eukaryotic Common Ancestor (LECA)

**Figure 1:**
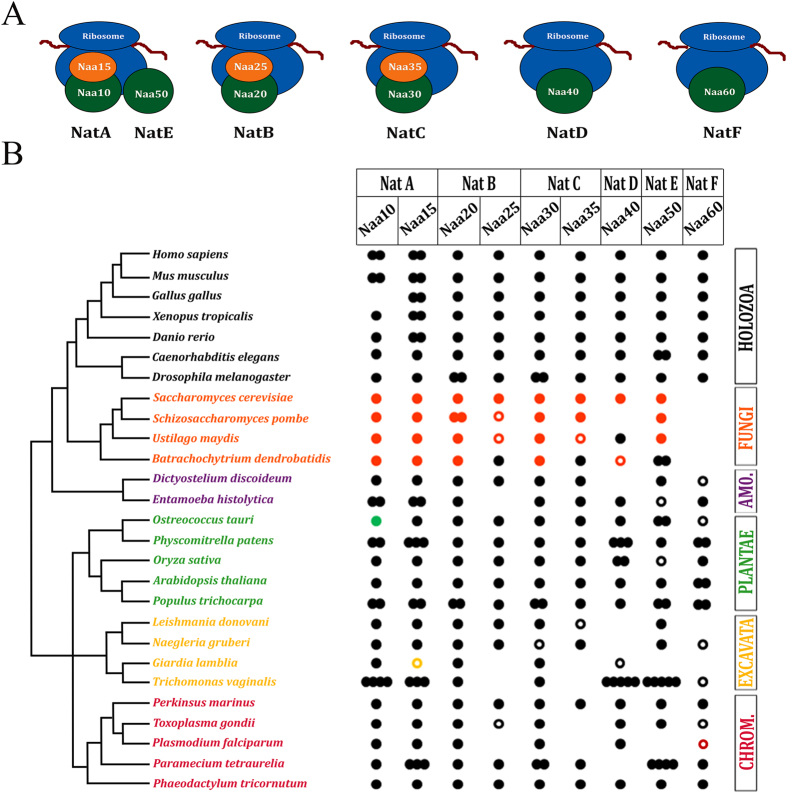
All six major human NAT complexes (NatA-F) were most likely present in the Last Eukaryotic Common Ancestor (LECA). (**A**) Subunits of all six major human NAT complexes (NatA-F). Catalytic subunits are shown in green, whereas regulatory subunits are shown in orange. (**B**) Catalytic and regulatory subunits of all six major human NATs complexes were identified across the eukaryotic tree of life, suggesting they were all present in the LECA. NATs subunit orthologs were identified in 27 species representative of the eukaryotic tree of life^59^,^60^,^61^,^62^,^63^. Naa60 (NatF) was apparently secondarily lost in fungi. Results are indicated according to reciprocal blastp E-value score (“filled dot” = E-value score lower than e^−8^; “open dot” = E-value score between e^−8^-e^−03^; “no dot” = E-value score higher than e^−03^. Black dot indicates NAT was identified using *H. sapiens* ortholog; orange dot indicates that NAT was identified using *S. cerevisiae* ortholog; green, yellow and red dots indicate that NATs were identified, respectively, using the phylogenetically closest plant, excavate and chromalveolata species ortholog. In the case of species-specific gene duplication, the number of dots is equivalent to the number of identified NAT paralogs. Phylogenetic distribution shown in this figure was previously reported^59^,^60^,^61^,^62^,^63^. Details of the original 73 analyzed eukaryotic species are shown in Supplementary Fig. 1 and Supplementary Table 1.

Since the number of distinct NAT complexes and N-terminally acetylated proteins increased in *H. sapiens* and *D. melanogaster* when compared to *S. cerevisiae* and prokaryotes^3^,^9^, we hypothesized that NATs had diversified during evolution of eukaryotic organisms. To test this hypothesis we selected 27 species representative of the major clades of eukaryotic tree of life: seven holozoa, four fungi, two amoebozoans, five plants, four excavates, and five chromalveolata. Bidirectional protein BLAST^57^ searches and HMMER^58^ gene model analyses were used to assess the presence or absence of the putative catalytic and regulatory subunits of the six major *H. sapiens* NAT complexes, or when necessary, NATs orthologs from more closely related species (e.g. *S. cerevisiae* for analysis of most fungi species). We considered NAT subunits orthologs on the basis of E-value of reciprocal best-hit in blastp (Fig. 1B; Supplementary Table 1). Detailed results for the originally analyzed 73 eukaryotic species are shown in Supplementary Fig. 1 and Supplementary Table 1.

Our analysis identified orthologs for the catalytic and regulatory subunits of all six NatA-F complexes (respectively, Naa10, Naa15, Naa20, Naa25, Naa30, Naa35, Naa40, Naa50, and Naa60) (Fig. 1A) in holozoa, amoebozoa, plantae, excavata, and chromalveolata (Fig. 1B). Consistent with our previous observation in *S. cerevisiae*^9^, we failed to detect Naa60 in the four fungi species (Fig. 1B). When considering the wide phylogenetic distribution of the analyzed eukaryotic species (Fig. 1B)^59^,^60^,^61^,^62^,^63^, we concluded that all major known *H. sapiens* NAT complexes were most likely present in the Last Eukaryotic Common Ancestor (LECA). Our results also suggested that Naa60 was secondarily lost in fungi (see below).

### Identified NATs are most likely catalytically active

**Figure 2:**
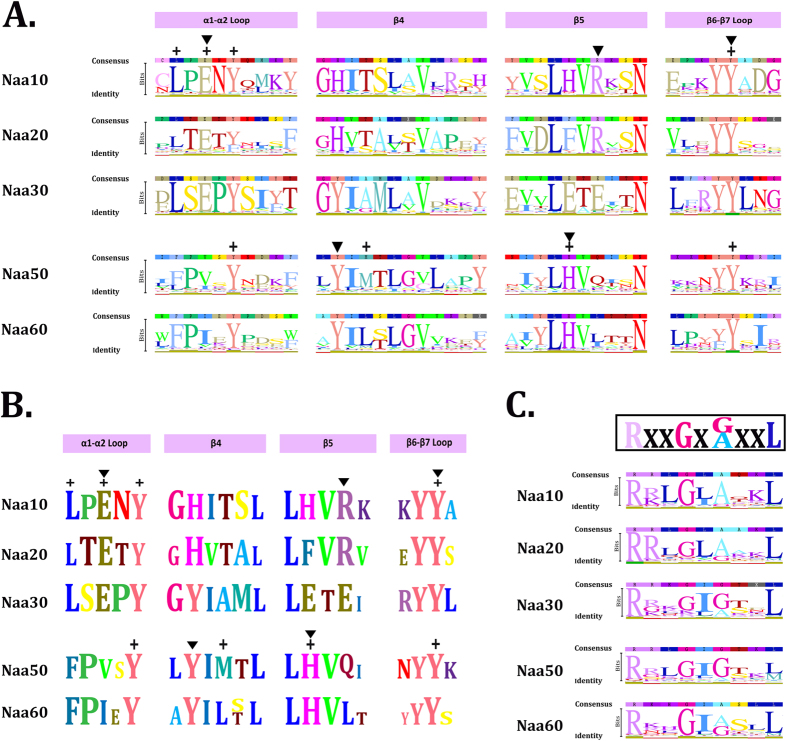
Identified NATs are most likely catalytically active. Identified NATs are most likely catalytically active as the acetyl-CoA binding domain^66^,^67^ and the catalytically active residues from *H. sapiens* NAT´s^64^,^65^ are highly conserved among these proteins. Protein sequences were retrieved from the genomes of 73 species representative of the eukaryotic tree of life and aligned. Letters height represents the degree of conservation of each amino acid residue for that position. The catalytically active residues (▼) and substrate binding residues (+) for Naa10 and Naa50 are indicated above each sequence alignment^64^,^65^. (**A** and **B**) The catalytically active residues of Naa10 (α1–α2 loop ‘E’; β5 helix ‘R’; β6-7 helix ‘Y’) and Naa50 (β4 helix ‘Y’; β5 helix ‘H’) are highly conserved among most identified NATs. The substrate binding residues for Naa10 (α1–α2 loop ‘L’, ‘E’, and ‘Y’; β6-7 helix ‘Y’) and Naa50 (α1–α2 loop ‘Y’; β4 helix ‘M’; β5 helix ‘H’; β6-7 helix ‘Y’) are also highly conserved among most identified NATs. The substrate binding and catalytically active residues of Naa10 are similarly highly conserved in Naa20 and Naa30. The only exception is the catalytically active arginine (R) in the β5 helix, which was replaced in Naa30 by a highly conserved glutamic acid (E) residue. The catalytically active glutamic acid (E) in α1–α2 loop and arginine (R) in the β5 helix are flanked by residues that vary specifically within Naa10, Naa20, and Naa30. (**C**) The acetyl coenzyme A binding motif, RxxGxG/A, which is highly conserved among enzymes of the N-acyltransferase superfamily^66^,^67^, is similarly conserved among most identified NATs.

If the identified NATs are catalytically active, the acetyl-CoA binding domain and catalytically active residues should be conserved among these proteins. To test this hypothesis we performed multiple sequence alignment of the protein sequences encoding the catalytic subunits of NATs, guided by the recently identified substrate-binding and catalytically active residues of *H. sapiens* Naa10 and Naa50^64^,^65^. Full protein alignment of distinct NAT catalytic subunit identified multiple highly conserved domains (data not shown). The acetyl coenzyme A binding motif, RxxGxG/A, is a sequence feature that is highly conserved among enzymes of the N-acyltransferase superfamily^66^,^67^ and is highly conserved in most NATs (Fig. 2C). The catalytically active residues of Naa10 (α1–α2 loop ‘E’; β5 helix ‘R’; β6-7 helix ‘Y’) and Naa50 (β4 helix ‘Y’; β5 helix ‘H’) (“inverted triangles” indicated on Fig. 2A,B)^64^,^65^, and the substrate binding residues for Naa10 (α1–α2 loop ‘L’, ‘E’, and ‘Y’; β6-7 helix ‘Y’) and Naa50 (α1–α2 loop ‘Y’; β4 helix ‘M’; β5 helix ‘H’; β6-7 helix ‘Y’) (“plus signs” indicated on Fig. 2A,B)^64^,^65^, were similarly highly conserved among most identified Naa10 and Naa50 orthologs.

In addition, all major functional domains of Naa10 and Naa50 (α1–α2 loop, β4, β5 and β6-7) were also highly conserved among Naa20, Naa30, and Naa60 (Fig. 2A,B). The only exception was Naa30, where the catalytically active arginine (R) in the β5 helix of Naa10 was replaced by a highly conserved glutamic acid (E) residue (Fig. 2A,B). Interestingly, the catalytically active glutamic acid (E) in α1–α2 loop and arginine (R) in the β5 helix were flanked by residues that varied specifically between Naa10, Naa20, and Naa30 (Fig. 2A,B). This confirmed that most identified NATs were correctly assigned to Naa10, Naa20, and Naa30, and suggested that this motif variation might be important for their distinct substrate specificities. Our results strongly suggest that most identified NATs are catalytically active N-terminal acetyltransferases. NATs whose canonical catalytically active residues vary from the *H. sapiens* orthologs are indicated in Supplementary Fig. 2.

### Clade-specific NAT loss during evolution of eukaryotic organisms

**Figure 3:**
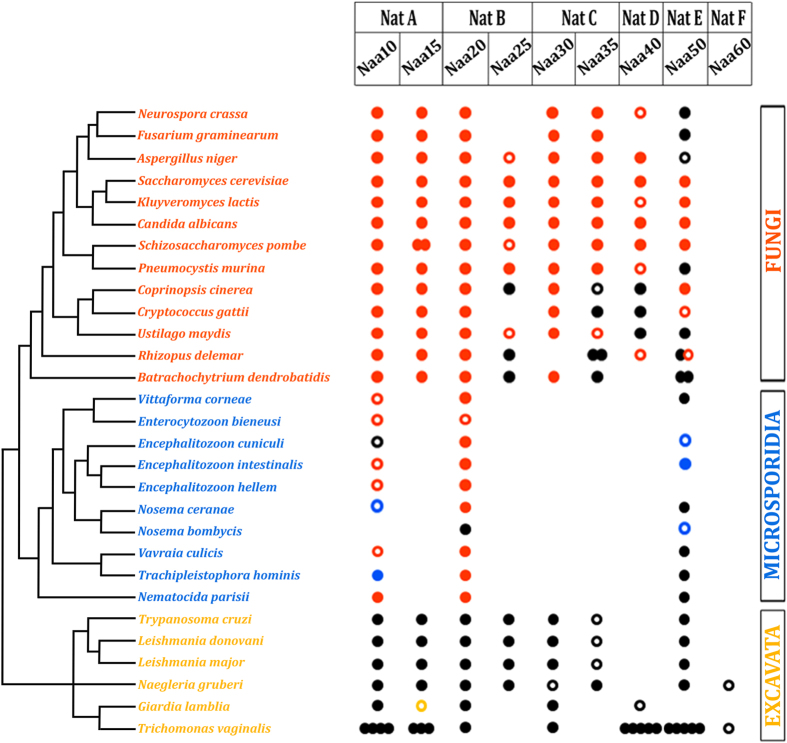
Clade-specific NAT loss during evolution of eukaryotic organisms. NAT complexes have been secondarily lost during evolution of major eukaryotic lineages. Naa10 and Naa20 were never or seldom lost during eukaryotic evolution (Fig. 1) (Supplementary Fig. 1), even in organisms like microsporidia with extensive gene loss^68^. Naa50 was comparatively more resilient to loss in distinct eukaryotic lineages (e.g. microspora and excavata) than Naa30, Naa40, and Naa60. Analysis of 13 species representative of the fungal kingdom shows that Naa60 is absent. The wide phylogenetic distribution of the fungal taxa suggests that Naa60 was most likely absent in the progenitor of all fungi. Results are indicated according to reciprocal blastp E-value score (“filled dot” = E-value score lower than e^−8^; “open dot” = E-value score between e^−8^-e^−03^; “no dot” = E-value score higher than e^−03^. Black dot indicates NAT was identified using *H. sapiens* ortholog; orange dot indicates that NAT was identified using *S. cerevisiae* ortholog; blue and yellow dots indicate that NATs were identified, respectively, using the phylogenetically closest microsporidia and excavate species ortholog. In the case of species-specific gene duplication, the number of dots is equivalent to the number of identified NAT paralogs. Phylogenetic distribution shown in this figure was previously reported^59^,^60^,^61^,^62^,^63^.

Although all major NAT complexes were most likely present in the LECA, we previously observed that Naa60 (the catalytic subunit of NatF) was absent in *S. cerevisiae*^9^. To test whether NatF is likely absent in all fungi, we analyzed 13 species representative of the fungal kingdom, and confirmed that Naa60 orthologs are absent (Fig. 3). The wide phylogenetic distribution of the analyzed fungal species suggests that Naa60 was most likely absent in the progenitor of all fungi.

We also investigated the genomes of species in the phylum microspora, as they are eukaryotic unicellular organisms and obligatory intracellular parasites that exhibit extreme genome reduction and gene loss^68^. We detected orthologs for Naa10, Naa20, and Naa50 in most microsporidia, yet we failed to identify orthologs for Naa30, Naa40, and Naa60 (Fig. 3). Loss of Naa40 and Naa60 was also observed within some but not all excavata (another large group of unicellular organisms) (Fig. 3). It is therefore evident that although all six NATs present in *H. sapiens* were most likely present in the LECA, some NATs have been lost during evolution of major eukaryotic lineages.

Surprisingly, in birds, we failed to identify Naa10 in galliformes (e.g. *Gallus gallus* (chicken) and *Meleagris gallopavo* (turkey)), passeriformes (e.g. *Taenopygia guttata* and *Ficedula albicollis*), and psittaciformes (e.g. *Melopsittacus undulatus* (budgerigar)) (Supplementary Fig. 4). However, given a) the extent of conservation of Naa10 across the eukaryotic tree of life (Fig. 1B; Supplementary Fig. 1), b) NatA’s broad role in protein Nt-acetylation, c) the identification of the Naa10-interacting subunit Naa15 (Supplementary Fig. 1; Supplementary Fig. 4), d) the identification of methionine aminopeptidases (MetAP) in all tested bird species (data not shown), and e) that Naa10 is present in some other birds (e.g. *Falco cherrug* (falcon) and *Anas platyrhynchos* (duck)) (Supplementary Fig. 1; Supplementary Fig. 4), we concluded that the absence of Naa10 in some birds is probably due to an unknown gap in the publicly available genome data. Undetectable Naa10 homologs due to rapid sequence divergence is not likely an explanation, as the protein sequences of the two identified birds’ Naa10 were highly conserved (Supplementary Table 1).

### NatE interacts and influences *in vivo* NatA catalytic activity

Naa10 and Naa20 were never or rarely lost during eukaryotic evolution (Fig. 1; Supplementary Fig. 1), even in organisms like microsporidia with extensive gene loss (Fig. 3)^68^, possibly because these two NATs Nt-acetylate the majority of the proteome^9^,^52^, likely making them indispensable. Surprisingly, Naa50 was comparatively more resilient to loss in distinct eukaryotic lineages than Naa30, Naa40, and Naa60 (Fig. 3; Supplementary Fig. 1).

**Figure 4:**
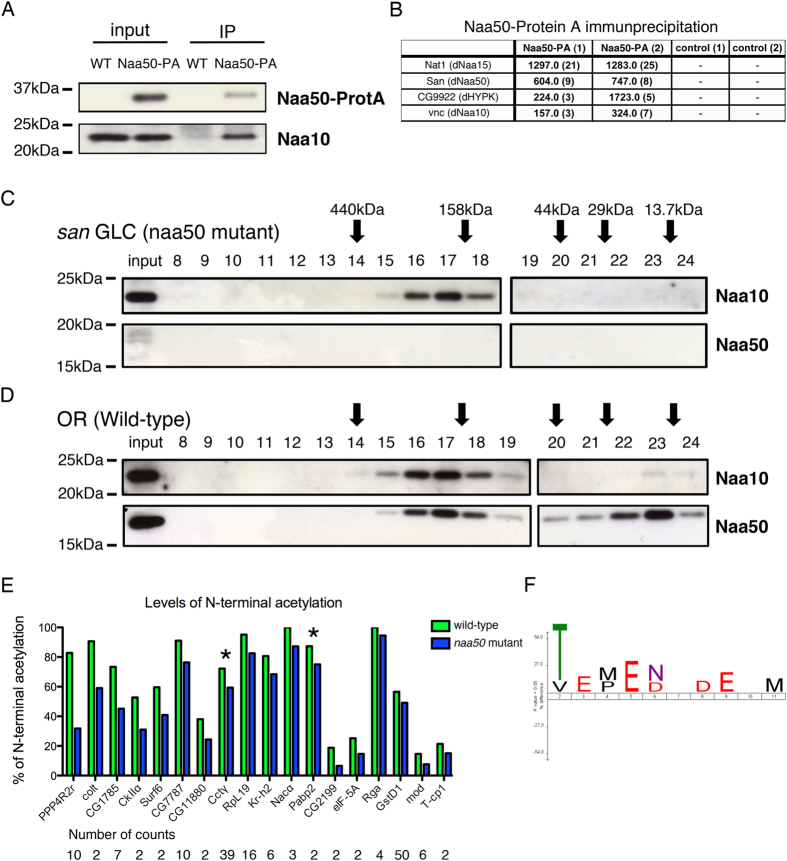
NatE interacts and influences *in vivo* NatA catalytic activity. *Drosophila* Naa50 is encoded by the gene separation anxiety (san). Naa50 physically interacts with NatA, and its loss influences the *in vivo* activity of NatA. (**A** and **B**). Naa50 (~20 kDa) physically interacts with NatA complex (Naa10 and Naa15) and HYPK/CG9922; respectively ~22 kDa, ~103 kDa, and ~14 kDa). Immunoprecipitated proteins were identified by western-blot (**A**) or by liquid chromatography coupled to tandem mass spectrometry (**B**). (**C** and **D**). Integrity of Naa10-containing complexes was apparently not affected after loss of Naa50. Size-exclusion chromatography of *san*^3^ mutant **(C)** and control **(D)** protein extracts from 0–2 h embryo collections. After separation, each fraction was analyzed by western blot. Naa10-containing complexes with approximately 150–350kDa were similarly identified in wild type and *san*^3^ mutant embryos (**D** versus **C**). There were significant levels of monomeric Naa50 (~20 kDa) in wild-type embryos (**D**). (**E** and **F**) Loss of Naa50 negatively influences the *in vivo* activity of NatA. The proteome of control and *san*^3^ maternal mutant embryos (0–2h collection) was analyzed by N-terminal COFRADIC^75^,^89^. (**E**) The N-terminal acetylation status of 265 unique N-termini was unequivocally determined, and the affected N-termini are shown for control (green bars) and *san*^3^ mutant embryos (blue bars). (**F**) Consensus sequence of affected N-termini suggested that most displayed a NatA-like substrate specificity (i.e. predominantly Thr-, Val-, Ser-, and Ala-starting N-termini). Only two of the affected N-termini started with a methionine (MF- and MQ-) (see asterisks indicated in panel E), which is consistent with the *H. sapiens* Naa50 substrate specificity^39^,^74^.

*Drosophila* Naa50 is encoded by the gene separation anxiety (san) and is required for sister chromatid cohesion and chromosome segregation during mitosis^69^,^70^. Consistently with previous reports^69^,^71^,^72^, Naa50 (~20kDa) physically interacted with NatA (Naa10 and Naa15; respectively ~22kDa and ~103kDa) (Fig. 4A,B). Naa10 and Naa50-containing complexes of approximately 150-350kDa were identified in wild-type *Drosophila* embryos (Fig. 4D), next to the detection of significant levels of monomeric Naa50. There was however no notable change in the apparent size of the Naa10-containing complexes in protein extracts from *san*^3^ mutant embryos (Fig. 4C versus 4D), which suggested that NatA complex integrity was not affected by loss of Naa50.

The NatA-interacting chaperone-like protein HYPK^73^ was also identified as a subunit of the immunoprecipitated Naa50-containing complex (Fig. 4B), suggesting that Naa50 interacts (directly or indirectly) not only with Naa10-Naa15 but also with a subset of other NatA-interacting proteins. This supports the hypothesis that the relative levels of ribosome associated Naa50/NatA and MetAPs (iMet-aminopeptidases) are important for the correct processing of the nascent polypeptide N-termini^74^ and further confirms the functional crosstalk between these two NAT complexes.

Since the physical interaction between NatA and NatE (Naa50) is conserved between *D. melanogaster* and *H. sapiens*, and in line with the recent observation that six canonical NatA substrates were less Nt-acetylated in *S. cerevisiae* lacking Naa50^74^, we hypothesized that Naa50 was infrequently lost during eukaryotic evolution because its absence partially inhibits NatA enzymatic activity. To further support this hypothesis, we performed N-terminal COmbined FRActional DIagonal Chromatography (COFRADIC) analysis^75^ on the proteomes isolated from control and *san*^3^ maternal mutant *Drosophila* embryos to assay their Nt-acetylomes, and to look for differences in N-terminal acetylation states. The *in vivo* N-terminal acetylation levels of 265 proteins were unequivocally determined in the proteomes of both control and *san*^3^ mutant embryos (Fig. 4E,F). 18 protein N-termini were identified as more Nt-acetylated in the control proteome expressing Naa50 as compared to the *san* mutant proteome (absence of Naa50). Consistent with our hypothesis, 16 out of 18 of the *san* mutant affected N-termini displayed NatA-like substrate specificity (Fig. 4E,F). Only two affected N-termini started with a methionine (MF- and MQ- starting N-termini) (see asterisks on Fig. 4E), the presumed *H. sapiens* Naa50 substrate specificity^39^,^74^. This suggests that loss of Naa50 in *D. melanogaster* embryos was associated *in vivo* with a reduction of NatA activity, which fully supports the hypothesis that Naa50 was infrequently lost during eukaryotic evolution because its absence partially impairs NatA enzymatic activity.

### No detectable proteome adaptation to NAT loss

NAT substrate specificity is largely defined by the identity of the substrate N-terminal second amino acid residue^6^,^9^,^39^,^48^,^49^,^50^,^51^,^76^. Since different NATs have distinct substrate specificities, and since these enzymes Nt-acetylate a large number of proteins, we tested whether the loss of NatC, NatD, and NatF in microsporidia was associated with a detectable decrease of their substrate N-termini when compared to NatA and NatB substrate N-termini. To investigate if there was such proteome-wide adaptation to NAT loss, we analyzed the amino acid frequency in N-terminal second, third, fourth, fifth, and sixth residue positions for 36 species representative of the eukaryotic tree of life, including 5 microsporidia species.

**Figure 5:**
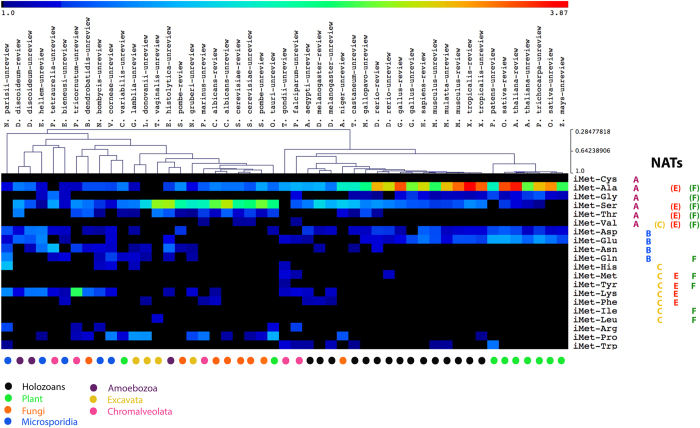
No detectable proteome adaptation to NAT loss. There are major biases in the frequency of amino acid usage at the N-terminal second position when compared to total proteome. However there is no detectable proteome adaptation to NAT loss. Loss of NatC and NatF in microsporidia (blue dots) and in some excavata (yellow dots) (Fig. 3) does not correlate with an increase usage frequency of NatA and NatB substrates. Comparatively to holozoans (black dots) and fungi (orange dots), microsporidia show an under-representation of alanine and serine residues (NatA substrates), no obvious over-representation of NatB substrates, and comparatively to higher eukaryotes, a moderate over-representation of lysine (NatC and NatE substrates). Amino acid usage frequency bias for each N-terminal position was analyzed by calculating the amino acid usage frequency for each position divided by its frequency in the total proteome (for more experimental detail see material and methods). The fold enrichment heat map shows the over-representation range (1 to 3.87 fold enrichment) of each amino acid at the N-terminal second position when compared to the total proteome. For each species and for each amino acid, it was attributed a black color (≤1.0) when the amino acid is under-represented in the N-terminal second position compared to its total proteome usage frequency. A detailed breakdown of the values used in this heat map is shown in Supplementary Table 2.

Previously, it was observed that the amino acid preference for the N-terminal second position varies, being lysine the most common amino acid residue in prokaryotes, serine the most common for lower eukaryotes, and alanine the most common for animals^9^,^52^,^77^,^78^. Consistently, we identified major biases in the frequency of amino acid usage at the N-terminal second position within distinct eukaryotic clades, including vertebrates and plants (Fig. 5; Supplementary Fig. 5; Supplementary Table 2). In vertebrates and plants, alanine residues (NatA substrate^6^,^7^,^50^) were significantly over-represented in the N-terminal second position when compared to the total frequency of usage of this amino acid in the proteome (Fig. 5; Supplementary Table 2), whereas serine residues (NatA substrate^6^,^7^,^50^) were significantly over-represented in the N-terminal second position of fungi and excavates (Fig. 5; Supplementary Table 2). Invertebrates showed instead an over-representation of both alanine and serine residues in the N-terminal second position (Fig. 5; Supplementary Table 2)^9^. Other amino acid residues like leucine, although commonly used in the entire proteome^52^, were nevertheless significantly under-represented at this position (data not shown). The N-terminal residue usage frequency biases were significantly less pronounced for the third, fourth, fifth and sixth N-terminal amino acid residues (Supplementary Fig. 5; Supplementary Table 2).

The N-terminal second residue position frequency bias observed in microsporidia does not support the hypothesis of a proteome-wide adaptation to loss of NatC, NatD, and NatF, with an increase frequency of NatA and NatB substrate N-termini. Compared to higher eukaryotes, microsporidia showed an under-representation of alanine and serine (NatA substrates), no obvious over-representation of NatB substrates, and a moderate over-representation of lysine (NatC and NatE substrates^9^,^23^,^25^,^39^,^76^). The moderate over-representation of lysine within these microorganisms is most likely not related to NAT loss, as the diatom *Phaeodactylum tricornutum* (chromalveolata), for example, showed an unusual over-representation of lysine within the N-terminal second position (Fig. 5) without NAT loss (Fig. 1). Some microsporidia, amoebozoa, chromalveolata, and excavata microorganisms also showed a moderate increase in the usage frequency of asparagine and glutamine residues (NatB substrates) in the N-terminal second position (Fig. 5). This over-representation is most likely also not related with NAT loss as *Paramecium tetraurelia* (chromalveolata), for example, showed a significant increase of these two residues (Fig. 5) without extensive NAT loss (Fig. 1). Consistently, differences in *S. cerevisiae* N-terminal sequences could not explain the absence of NatF in this unicellular organism^9^.

We concluded that although the N-terminal second residue position is under significant clade-specific constraints when compared to other nearby residues, there is no evidence supporting the hypothesis of a proteome-wide adaptation of proteins N-termini to NAT loss. The extensive loss of NATs might be accommodated by an increased substrate redundancy of the remaining NATs in microsporidia. Alternatively, it is also possible that Nt-acetylation is not functionally rate limiting for most proteins.

## Discussion

The origin of eukaryotes is still poorly understood. The organizational complexity of a eukaryotic cell is significantly higher than any known prokaryote. Eukaryotic cell compartmentalization is supported by an elaborate endomembrane system and by an actin/tubulin-based cytoskeleton. Conservation of the major features of eukaryotic cell organization and of a large set of genes demonstrates that they were already present in the Last Eukaryotic Common Ancestor (LECA)^79^. Protein Nt-acetylation is an ancient and highly conserved protein modification. Although the number of NAT complexes significantly increased in eukaryotic cells when compared to prokaryotic organisms, our work demonstrates that all major human NAT complexes diversified before the evolution of eukaryotic organisms, as they were already present in LECA.

Clade-specific NAT duplications exist across the eukaryotic tree of life^54^,^55^,^56^. In mammals, gene duplications of both Naa10 and Naa15 resulted in Naa11 and Naa16 paralogs, respectively^54^,^55^. Both gene paralogs encode proteins that are likely to participate in functional NatA complexes. Although human Naa11 and Naa16 share 81% and 70% sequence identity with Naa10 and Naa15, respectively^54^,^55^, Naa16 expression is particularly high on certain tissues like adrenal gland, mammary gland, heart, thymus, and testis^55^. Expression of mouse Naa11 is similarly restricted, being particularly high during spermatogenesis^80^. Since mouse Naa10 locates on chromosome X, Naa11 expression is likely to have a compensatory function after the loss of Naa10 expression (approximately in 50% of the sperm) during meiosis. Therefore, and although the increased complexity of the higher eukaryotic proteome clearly occurred without a concomitant diversification of the major known NAT complexes, clade-specific gene duplications likely happened to address specific functional requirements.

It is possible to argue that the NAT substrate repertoire has not been significantly increased during eukaryotic evolution, as these enzymes mostly rely on the identity of the substrate N-terminal second residue for specificity. Nevertheless, and next to purely housekeeping functions for Nt-acetylation, the regulatory complexity of this co-translational modification is likely to have increased in higher eukaryotes as their proteome and cellular behavior became more complex. Consistently, detectable co-evolution between NATs substrate specificities and the eukaryotic proteome has recently been reported in humans for NatA and NatD^47^,^81^. In the absence of NAT diversification, we speculate that transcriptional and post-transcriptional regulation of NAT activity are likely to play increasingly important roles in the regulation of Nt-acetylation during development of multicellular organisms^82^.

## Material and Methods

### Identification of NAT orthologs

Reference protein sequences of NAT complexes subunits (Naa10, Naa15, Naa20, Naa25, Naa30, Naa35, Naa40, Naa50 and Naa60) in *H. sapiens* and *S. cerevisiae* were obtained through literature mining^9^,^83^. These sequences, or sequences from closely related species, were used to identify putative orthologs from 73 species (Supplementary Fig. 1) representative of the eukaryotic tree of life^59^,^60^,^61^,^62^,^63^. Publicly available genome databases (e.g. NCBI and Ensembl) were used to retrieve these sequences. Two steps were used to verify the identity of these putative orthologs: we used reciprocal bidirectional protein BLAST (blastp)^57^. The retrieved proteins were only considered *bona fide* orthologs if they corresponded to the blastp best hit in both directions. The results were subjectively divided in three classes: “filled dot”: reciprocal blastp E-value score lower than e^−8^; “empty dot”: reciprocal blastp E-value score between e^−8^- e^−03^; “no dot”: reciprocal blastp E-value scores higher than e^−03^ (Fig. 1B, Fig. 3, Supplementary Fig. 1–4, Supplementary Table 1). To identify apparently missing ortholog sequences, additional searches were performed using protein sequence queries from phylogenetically closely related organisms. For example, *Populus trichocarpa* (seed plant) protein sequences were used for ortholog identification in the closely related (in the context of eukaryotes) *Osteococcus tauri* (green algae). To confirm the bidirectional blastp results, we built a separate hidden markov model for each NAT protein using HMMER 3.0^58^. Multiple sequence alignments of ortholog protein sequences positively identified by reciprocal BLAST searches were used to calculate hidden markov models from GenBank non-redundant (nr) protein database (downloaded August 2013). These HMM profiles allowed us to review the fit of the sequence to our orthologue classification.

### Sequence alignment and functional domain arrangement

For the identification of conserved domains within NAT catalytic subunits all identified orthologs of Naa10, Naa20, Naa30, Naa50, and Naa60 were retrieved from 73 eukaryotic species and aligned using ClustalW^84^ program in the Geneious software (version 6.1.8) with default values. Sequence alignments were manually edited by removing ambiguously aligned sequences and gaps in Gblocks^85^. Regions in the alignment with more than 50% gaps were removed. Four domains α1–α2 loop, β4 helix, β5 helix and β6-7 helix were previously shown to be catalytically important in NATs^64^,^65^. These highly conserved domains were used to validate the alignment. The three catalytically active residues of Naa10 (α1–α2 loop ‘E’, β5 helix ‘R’ and β6-7 helix ‘Y)’ were identified and marked as (▼) (Fig. 2A,B). Substitution of the canonical catalytically active residues for alternative residues is shown in Supplementary Figs 2 and 3. The two catalytically active residues of Naa50 (β4 helix ‘Y’ and β5 helix ‘H’) were marked as (▼) (Fig. 2A,B). The acetyl coenzyme A (AcCoA) binding motif^66^,^67^, RxxGxG/A in NATs was manually assigned in the aligned sequences (Fig. 2C).

### Fly work and genetics

*Drosophila melanogaster* flies were raised using standard techniques^86^. *san*^3^ mutant allele was previously reported as a loss-of-function allele of the gene separation anxiety (san)^70^, which encodes *Drosophila* Naa50. Maternal mutant embryos of *san*^3^ were generated using the FLP/FRT ovo^D^ system^87^.

The Naa50 open-reading frame (from cDNA clone AT27602) was subcloned into a vector containing the UASp promoter and C-terminal Protein A-tag (Gateway, Life Technologies). All constructs were then used to generate transgenic fly stocks (BestGene).

### Protein extracts

Collections of 0–2 hr (after egg-laying) *Drosophila* embryo were washed in PBS + 0.1% tween 20, dechorionated with 50% of bleach (commercial solution) for 5 minutes, and thoroughly washed in water. Protein extraction was performed through homogenization of embryos in NB lysis buffer (150 mM NaCl, 50 mM Tris-HCl pH 7.5, 2 mM EDTA, 0.1% NP-40), 1 mM DTT, 10 mM NaF, and EDTA-free protease inhibitor cocktail (Roche, Germany). Extracts were centrifuged at 4 °C and 21,100 g for 3 minutes. Supernatant was collected (being the upper lipid-rich layer avoided as much as possible) and centrifuged twice more. Total protein concentration was determined using Bio-Rad Bradford protein assay (BioRad, Hercules, CA, USA).

### Antibodies

Primary antibodies used were rabbit anti-San/Naa50 (1:1000)^69^ and rabbit anti-Ard1/Naa10 (1:2000) (Santa Cruz; FL-235)^88^. Secondary detection in western-blot was performed with rabbit HRP-conjugated antibodies (Jackson ImmunoResearch) used at a final concentration of 1:5000.

### Co-Immunoprecipitation

Total proteins extracts of *Drosophila* embryos expressing Naa50/San fused to a C-terminal Protein A-tag (Naa50/San-PA) were obtained. Dynabeads M-270 Epoxy (Invitrogen, Grand Island, NY, USA) pre-incubated with rabbit IgG immunoglobulins (5 μg IgGs/ 1 mg of beads) (Dynabeads antibody coupling kit, 14311D, Invitrogen), were added to 1.5 mg of protein extract and incubated for 1 hour at 4 °C. After washing the beads 3 times with NB buffer, protein elution was performed with 100 μl of 100 mM Glycine pH 3.0 during 1 minute and stopped with 10 μl of 1 M Tris Base pH 10.8. Eluted proteins were precipitated at −20 °C using 5 volumes of acetone. Precipitated samples were analyzed by liquid chromatography coupled to tandem mass spectrometry (Mass Spectrometry Laboratory, Institute of Biochemistry and Biophysics, Poland). For western-blot analysis eluted proteins were boiled in SDS-sample buffer for 5 minutes.

### Size exclusion chromatography

Size-exclusion chromatography was performed by applying protein extracts of 0–2 h collections from FRT42B *san*^3^ mutants or control (Oregon R) *Drosophila* embryos. Extracts were prepared in NB2 buffer (150 mM NaCl, 50 mM Tris-HCl pH 7.5, 2 mM EDTA, 0.01% NP-40), 1 mM DTT and EDTA-free protease inhibitor cocktail (Roche). Subsequently, 2 mg of protein extract were fractionated using Superose 6 10/300 GL column (GE Healthcare) in NB2 buffer and analyzed by western blot using antibodies against Naa10/Ard1^88^ and Naa50/San.

### Mass spectrometry

Liquid chromatography coupled to tandem mass spectrometry was performed at the Mass Spectrometry Laboratory (Institute of Biochemistry and Biophysics, Poland).

Briefly, peptides mixtures were analyzed by LC-MS-MS/MS (liquid chromatography coupled to tandem mass spectrometry) using Nano-Acquity (Waters, Milford, MA, USA) LC system and Orbitrap Velos mass spectrometer (Thermo Electron Corp., San Jose, CA, USA). Prior to analysis, proteins were subjected to standard ‘in-solution digestion’ procedure, during which proteins were reduced with 100 mM DTT (for 30 minutes at 56 °C), alkylated with 0.5 M iodoacetamide (45 minutes in darkroom at room temperature), and digested overnight with trypsin (Sequencing Grade Modified Trypsin-Promega V5111). The peptide mixture was applied to an RP-18 precolumn (nanoACQUITY Symmetry C18-Waters 186003514) using water containing 0.1% TFA as mobile phase, then transferred to nano-HPLC RP-18 column (nanoACQUITY BEH C18-Waters 186003545) using an acetonitrile gradient (0%–35% AcN in 180 min) in the presence of 0.05% formic acid with a flow rate of 250 nl/min. The column outlet was directly coupled to the ion source of the spectrometer, operating in the regime of data dependent MS to MS/MS switch. A blank run ensuring no cross contamination from previous samples preceded each analysis.

Raw data were processed by Mascot Distiller followed by Mascot Search (Matrix Science, London, UK, on-site license) against Flybase database. Search parameters for precursor and product ions mass tolerance were 15 ppm and 0.4 Da, respectively, enzyme specificity: trypsin, missed cleavage sites allowed: 0, fixed modification of cysteine by carbamidomethylation, and variable modification of methionine oxidation. Peptides with Mascot Score exceeding the threshold value corresponding to <5% false positive rate, calculated by Mascot procedure, and with the Mascot score above 30 were considered to be positively identified.

### N-terminal COFRADIC analysis

The proteome of control (Oregon R) and *san*^3^ maternal mutant *Drosophila* embryos (0–2 h collection) were analyzed by N-terminal COFRADIC analysis as described previously^75^,^89^. Overall, 432 unique N-termini derived from 399 unique SwissProt *Drosophila* protein accessions were identified. N-termini that partially retain their initiator-Met and/or have most probably alternative or miss-annotated translation products led to the higher number of unique N-termini as compared to their corresponding identified accession. Quantification of the degree of Nt-acetylation was performed as described^9^. A significant variation in the degree of Nt-acetylation was set to 10% or more (p ≤ 0.01). Further, N-termini were considered to be affected by *san* deletion that were fully acetylated in the control strain and displayed a reduction of more than 5% in the *san* deletion strain were also considered.

As such, the N-terminal acetylation states of the N-termini identified in both setups (292 unique N-termini) were comparatively analyzed. In this study we distinguished between *in vivo* N-terminal acetylation and free N-termini by making use of *in vitro* AcD3C13-acetylation (i.e. 5Da heavier form of NHS-acetate which introduces an 13C2 and D3 labeled acetyl moiety on all free amines).

An N-terminus is defined as 1) a peptide that is *in vivo* N-terminal acetylated or *in vitro* AcD3C13-acetylated peptide (i.e. an *in vivo* free N-terminus) and starts in the Swiss-Prot database at position 1 or 2 of the annotated protein sequence, or 2) an *in vivo* N-terminal acetylated peptide, with a protein sequence starting position beyond position two. Only peptides displaying an internal start position and of which at least one N-terminal acetylated peptide was identified in one of the two setups analyzed were considered (i.e. internal AcD3C13-acetylated peptides were not considered in this study).

Overall the N-terminal acetylation levels of 265 out of the 292 unique N-termini identified in both setups could be unequivocally determined in control as well as the *san* mutant setup, and for the affected N-termini, the difference (∆) in the N-terminal acetylation status of the control versus *san* mutant setup was analyzed and is shown (Fig. 4E). In total, 10 unique N-termini (start 1/2) and 4 N-termini (internal start position) displayed in san mutant embryos a decrease of at least 10% in the overall N-terminal acetylation. 12 out of 14 (or 87%) of the *san* mutant affected N-termini displayed NatA-like substrate specificity (i.e. predominantly Thr (8/14 or 57%), Ser, Val and Ala-starting N-termini (Fig. 4F). Two affected N-termini started with a methionine (MF- and MQ- starting N-termini) (see “stars” in Fig. 4E). 4 unique N-termini displayed a decrease of more than 5% (but less than 10%) in the overall N-terminal acetylation. All four N-termini displayed NatA-like substrate specificity. LC-MS/MS analysis, data processing and storage were performed as described^9^.

### Whole proteome sequences and frequency analysis

We selected 38 species (ten holozoa, seven plant, five fungi, five microsporidia, four excavata, five chromalveolata, and two amoeboza) to cover the full range of eukaryotic proteome complexity. Whole datasets of proteins from these organisms with complete proteome annotations available in UniProt were downloaded as FASTA formats in April 2015 (http://www.uniprot.org). We used reviewed (UniProtKB/Swiss-Prot) and unreviewed (UniProtKB/TrEMBL) protein entries for each analyzed species. Yet, if the reviewed proteome had less than thousand proteins than only the more extensive unreviewed proteome was used.

We analyzed the amino acid residue frequency bias (over or under-representation) for each N-terminal position by calculating the residue frequency for each N-terminal position divided by the total proteome frequency of this residue (program script for N-termini analysis is available upon request). MultiExperiment Viewer (MeV), which is part of the TM4 Microarray Software Suite (v4.9), was used to perform hierarchical clustering and to generate heat maps with frequency of amino acid at second, third, fourth fifth, and sixth position^90^. The parameters used for the hierarchical clustering were the sample tree, Pearson correlation and the average linkage method.

## Additional Information

**How to cite this article**: Rathore, O. S. *et al.* Absence of N-terminal acetyltransferase diversification during evolution of eukaryotic organisms. *Sci. Rep.* **6**, 21304; doi: 10.1038/srep21304 (2016).

## Supplementary Material

Supplementary Information
