# Supplementary material for: Absence of N-terminal acetyltransferase diversification during evolution of eukaryotic organisms
Source: Sci Rep. 2016 Feb 10;6:21304. doi: 10.1038/srep21304 (PMC4748286; doi:10.1038/srep21304)
Supplement: Supplementary Information [file srep21304-s1.pdf]

Absence of N-terminal acetyltransferase diversification during evolution of eukaryotic organisms.

Om Singh Rathore[1,2,3], Alexandra Faustino[1,2], Pedro Prudêncio[1,2,5], Petra Van Damme[6,7], Cymon J. Cox[4], Rui Gonçalo Martinho[1,2,5,#].

[1]Department of Biomedical Sciences and Medicine, [2]Center for Biomedical Research (CBMR), [3]ProRegeM - PhD Program in Mechanisms of Disease and Regenerative Medicine, [4]Center of Marine Sciences, University of Algarve, Faro, Portugal; [5]Instituto Gulbenkian de Ciência, Rua da Quinta Grande 6, Oeiras 2781-901, Portugal; [6]Department of Medical Protein Research, VIB, B-9000 Ghent, Belgium; [7]Department of Biochemistry, Ghent University, B-9000 Ghent, Belgium

# To whom correspondence should be addressed:
Email: rgmartinho@ualg.pt

Supplementary Figure 1

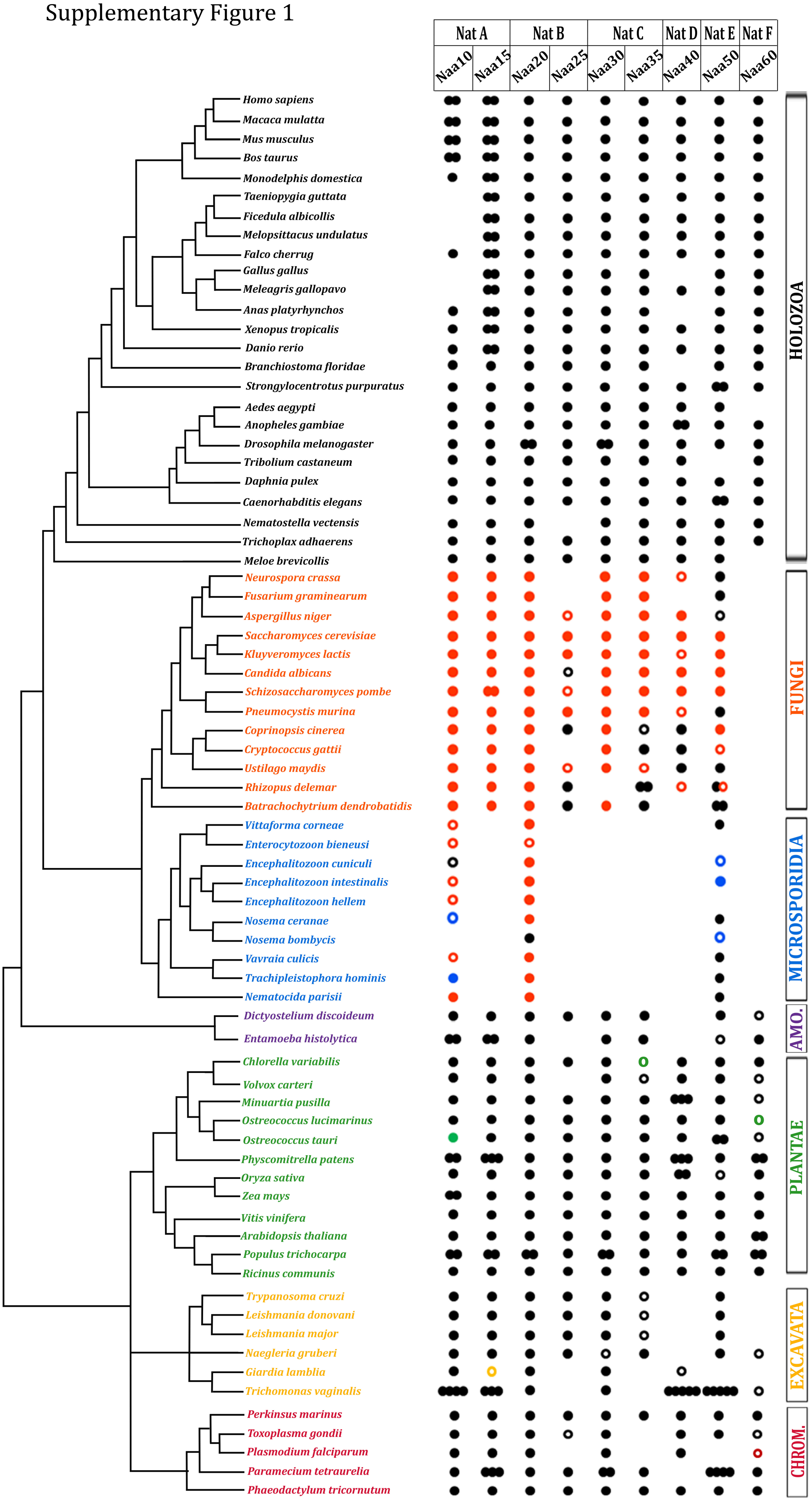

Supplementary Figure 2

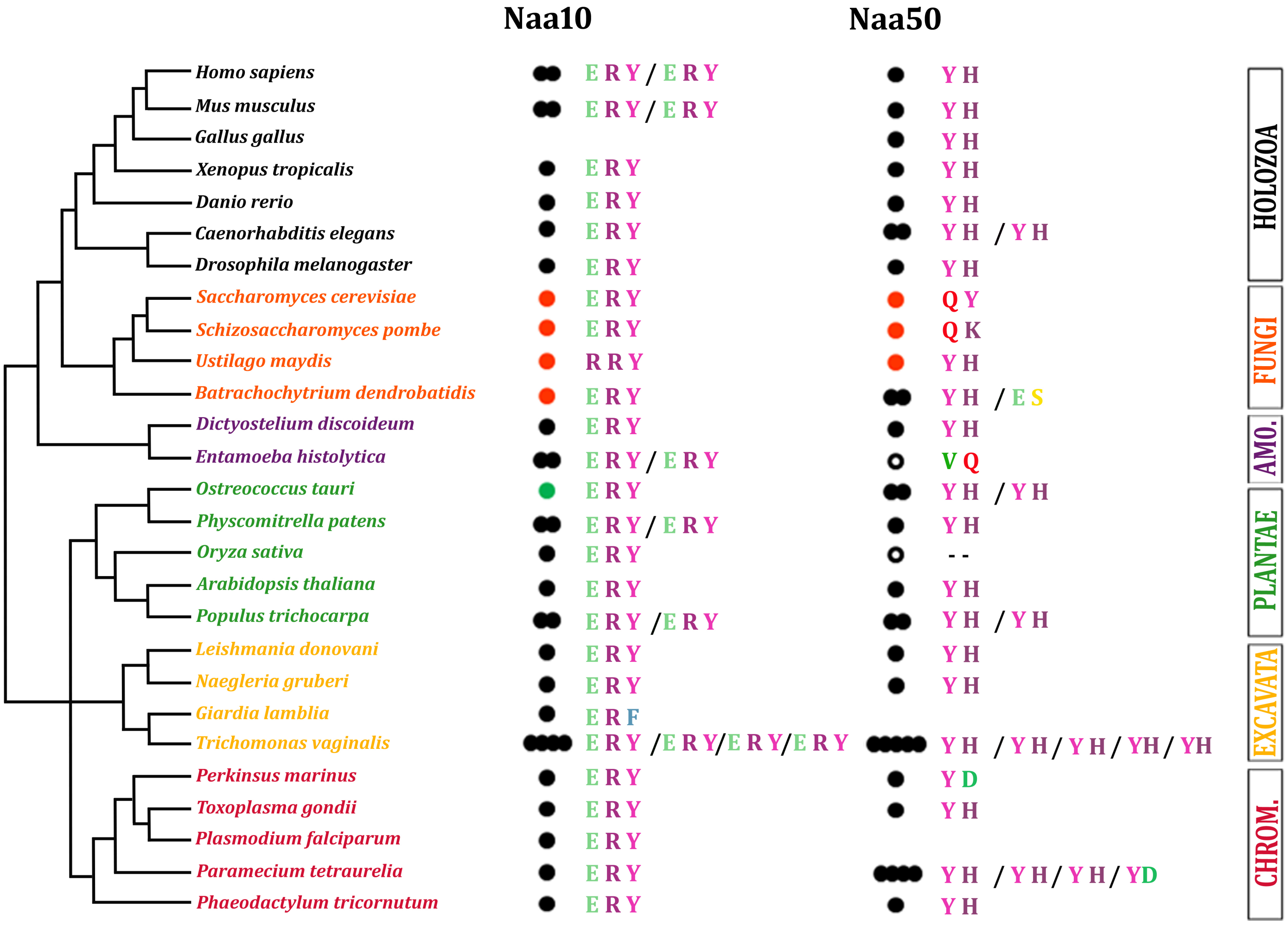

Supplementary Figure 3

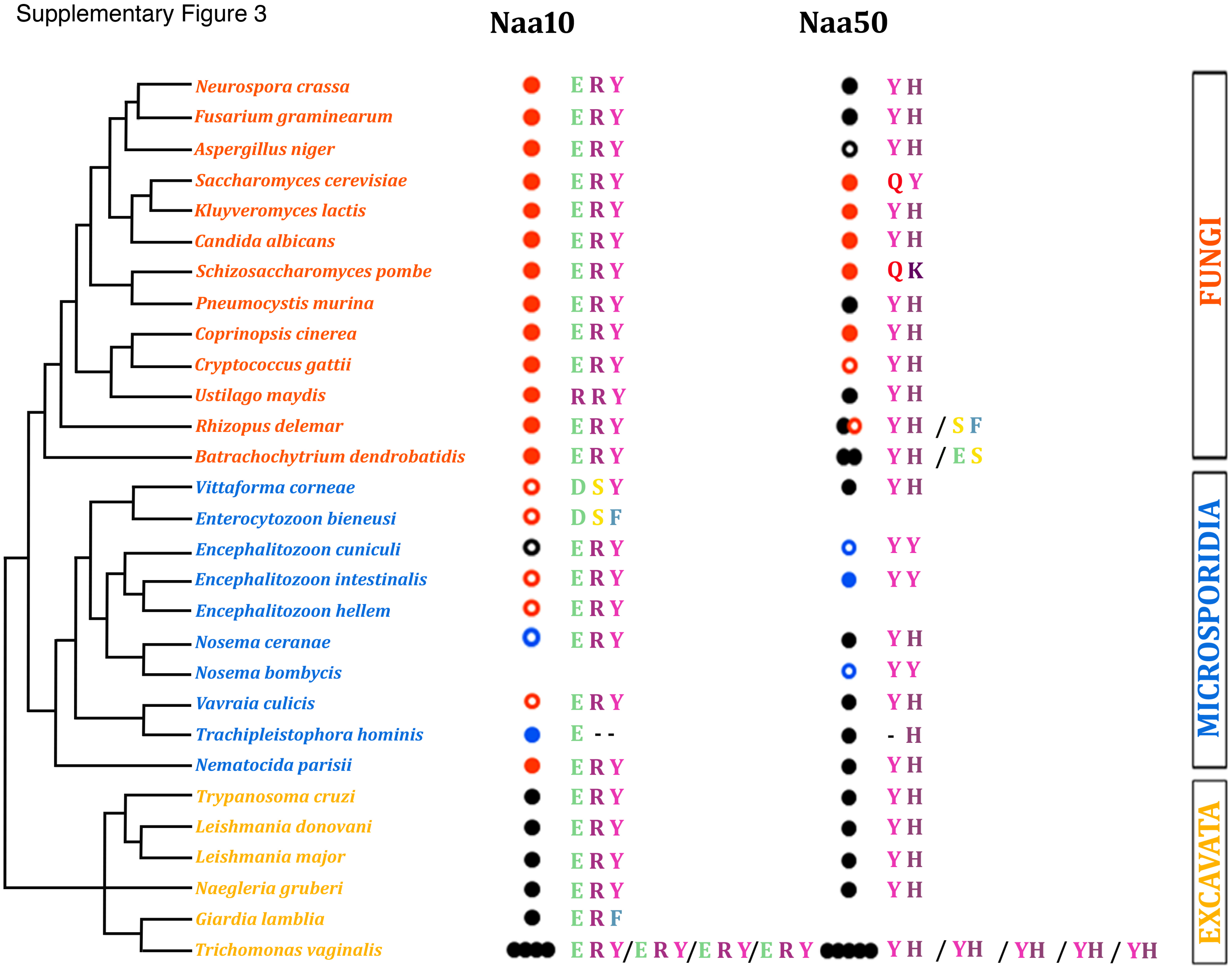

Supplementary Figure 4

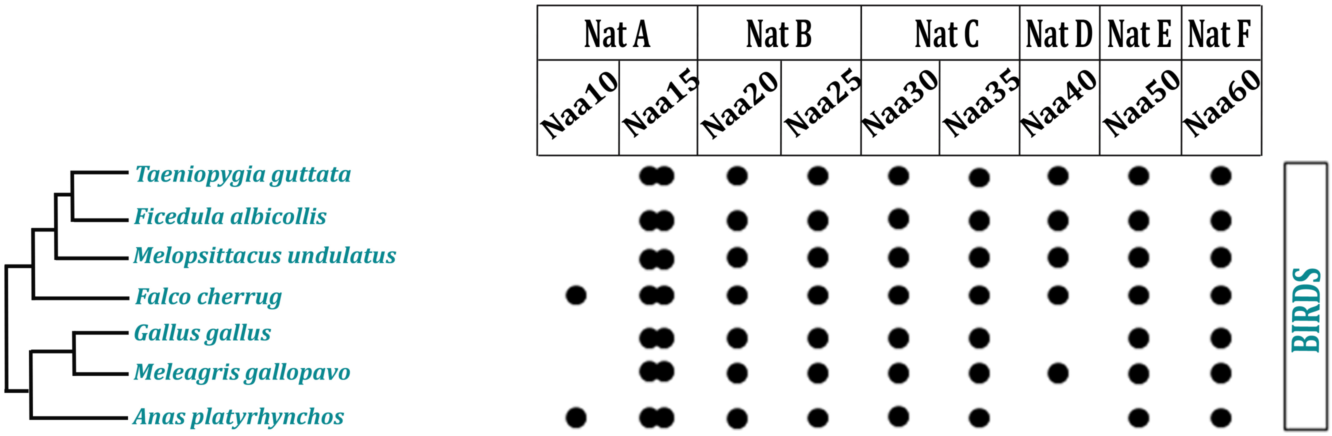

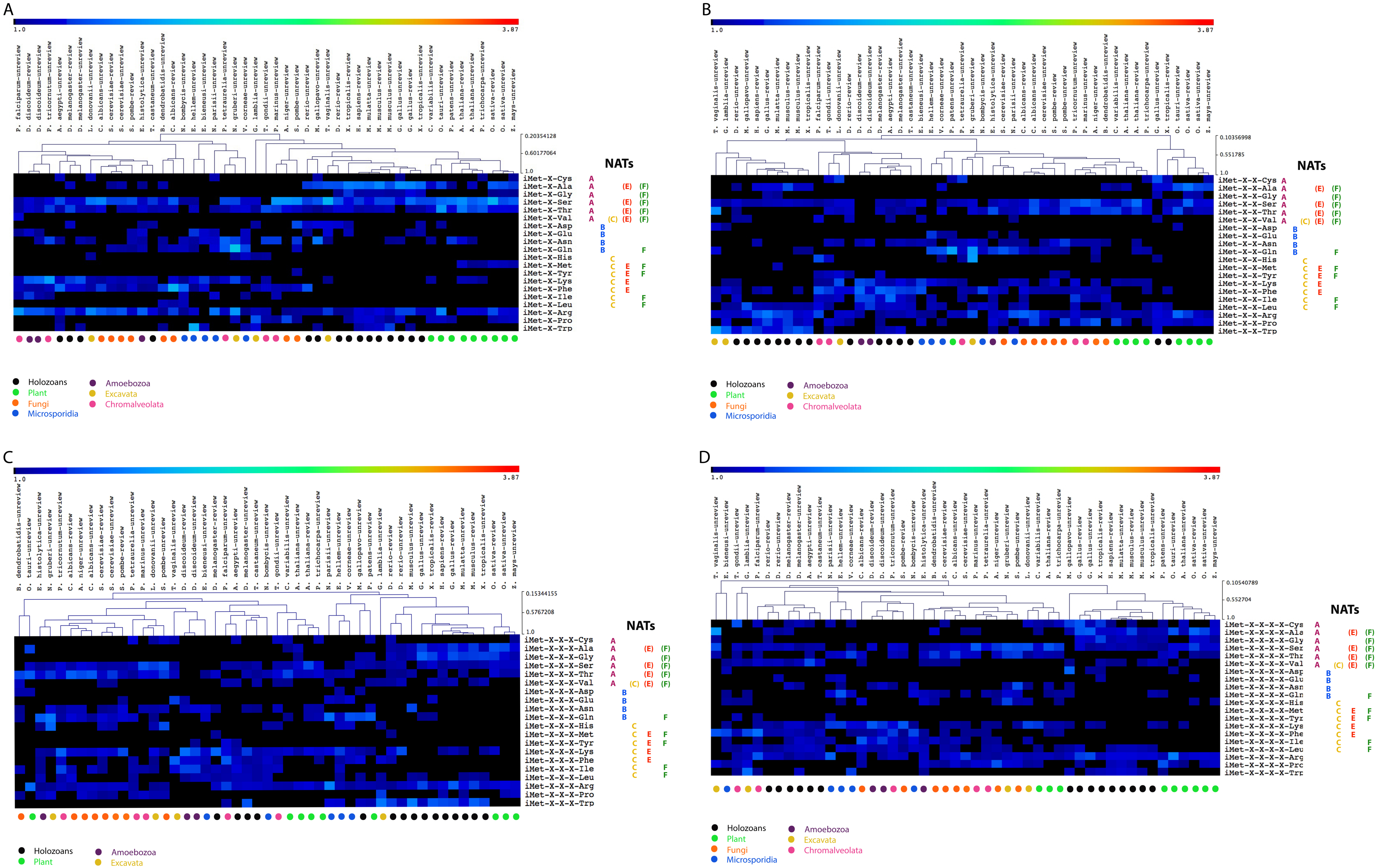

Supplementary Figure 5

Supplementary Table 1

	Nat A		Nat B		Nat C		Nat D	Nat E	Nat F
	**Naa10/11**	**Naa15/16**	**Naa20**	**Naa25**	**Naa30**	**Naa35**	**Naa40**	**Naa50**	**Naa60**
**HOLOZOA**									
Homo sapiens	NP_003482.1/<br>NP_116082.1	NP_476516.1/<br>NP_078837.3	NP_057184.1	NP_079229.2	NP_001011713.2	NP_078911.3	NP_079047.2	NP_079422.1	NP_079121.1
Macaca mulatta	XP_001089324.2/<br>NP_001180830.1	NP_001245032.1/<br>NP_001253545.1	XP_001091322.2	XP_001102995.2	NP_001253809.1	NP_001181318.1	XP_001118319.2	NP_001248300.1	NP_001253671.1
	1e-158/3e-170<br>2E-165/3e-158	0/0<br>0/0	6e-94/3e-93	0/0	0/0	0/0	7e-153/2e-177	3E-124/1e-123	0/1e-179
Mus musculus	NP_063923.1/<br>NP_001028363.1	NP_444319.3/<br>NP_080108.1	NP_001135437.1	NP_766310.2	NP_001074899.1	NP_084429.2	NP_081919.1	NP_082384.1	NP_083366.1
	1e-161/2e-161<br>6E-137/4e-132	0/0<br>0/0	4e-131/7e-131	0/0	2e-169/4e-172	0/0	2e-178/3e-178	7e-123/1e-122	3e-177/5e-177
Bos taurus	NP_001039976.1/<br>NP_001193340.1	NP_001178248.1/<br>NP_001096570.1	NP_001193758.1	NP_001193056.1	NP_001192966.1	NP_001180040.1	NP_001092474.1	NP_001069218.1	NP_001069117.1
	1E-165/7e-165<br>4E-155/4e-149	0/0<br>0/0	2e-131/7e-131	0/0	0/0	0/0	1e-177/6e-177	3e-124/1e-123	8e-175/3e-174
Monodelphis domestica	XP_007507105.1	XP_001377801.1/<br>XP_001368680.2	XP_001374084.1	XP_001365908.1	XP_003339539.1	XP_003341702.1	XP_001368911.2	XP_001367592.1	XP_001370249.1
	1e-146/7e-148	0/0<br>0/0	7e-131/5e-130	0/0	4e-162/3e-162	0/0	3e-169/8e-169	3e-120/2e-119	2e-172/2e-171
Taeniopygia guttata	-/-	XP_002191713.2<br>/XP_002196302.1	NP_001232487.1	XP_002193478.2	XP_002200419.2	XP_002191777.1	XP_012432286.1	XP_002186676.1	XP_002195683.1
		0/0<br>0/0	5e-130/6e-129	0/0	8e-124/1e-124	0/0	1e-87/8e-85	4e-121/4e-120	1e-169/1e-168
Ficedula albicollis	-/-	XP_005044911.1/<br>XP_005037896.1	XP_005050761.1	XP_005055325.1	XP_005047886.1	XP_005061005.1	XP_005062675.1	XP_005038535.1	XP_005054206.1
		0/0<br>0/0	7e-130/1e-75	0/0	2e-125/1e-124	0/0	3e-73/9e-78	1e-118/9e-118	1e-168/9e-168
Melopsittacus undulatus	-/-	XP_005142434.1 /<br>XP_005145707.1	XP_005141365.1	XP_005145220.1	XP_005152409.1	XP_005154988.1	XP_005140525.1	XP_005143088.1	XP_005152549.1
		0/0<br>0/0	3e-128/3e-127	0/0	1e-118/2e-122	0/0	5e-54/1e-53	1e-117/8e-116	5e-169/ 4e-167
Falco cherrug	XP_005447033.1	XP_005439245.1<br>/XP_005433302.1	XP_005437001.1	XP_005436785.1	XP_005437971.1	XP_005441431.1	XP_005435899.1	XP_005433422.1	XP_005436827.1
	6e-111/1e-115	0/0<br>0/0	4e-104/4e-114	0/0	7e-115/1e-122	0/0	1e-96/2e-121	1e-119/2e-118	4e-170/5e-169

Anas platyrhynchos	EOA93076.1	XP_005022466.1/<br>XP_005037896.1	XP_005031564.1	XP_005030368.1	XP_005020924.1	XP_005017302.1	-/-	XP_005021905.1	XP_005028567.1
	1e-24/1e-24	0/0<br>0/0	8e-25/6e-25	0/0	1e-119/2e-125	0/0		3e-118/2e-117	2e-169/1e-168
Gallus gallus	-/-	XP_420407.4/<br>XP_417028.2	XP_004943128.1	NP_001025835.2	XP_004936526.1	NP_001026623.1	XP_003643576.2	NP_001025949.1	XP_414956.1
		0/0<br>0/0	1e-132/4e-133	0/0	3e-119/2e-125	0/0	4e-154/2e-153	2e-112/2e-109	2e-168/4e-171
Meleagris gallopavo	-/-	XP_003205449.1/<br>XP_003203369.1	XP_003209661.1	XP_003211005.1	XP_003206855.1	XP_003213623.1	-/-	XP_003202741.1	XP_003210809.1
		0/0<br>0/0	3e-116/4e-115	0/0	2e-111/1e-118	2e-123/3e-123		8E-119/2e-117	5e-169/7e-168
Xenopus tropicalis	NP_001007497.1	NP_001017001.2/<br>XP_002938092.2	NP_989110.1	XP_004910562.1	XP_002933772.1	NP_001186425.1	NP_001120405.1	NP_001011131.1	NP_001106444.1
	4e-138/1e-137	0/0<br>0/0	9e-130/5e-129	0/0	2e-118/6e-117	0/0	4e-147/1e-146	2e-117/1e-116	1e-149/6e-149
Danio rerio	NP_998499.1	NP_956940.1/<br>NP_976066.1	NP_001014351.1	XP_005172655.1	NP_001129721.2	NP_955844.1	NP_001014351.1	XP_005172655.1	NP_001129721.2
	1e-126/1e-123	0/0<br>0/0	8e-131/4e-130	0/0	2e-105/4e-106	0/0	8e-131/4e-130	0/0	2e-105/4e-106
Branchiostoma floridae	XP_002594750.1	XP_002611320.1	XP_002610035.1	XP_002599541.1	XP_002597154.1	XP_002601534.1		XP_002610693.1	XP_002595762.1
	1e-70/1e-69	0/0	5e-108/3e-107	0/0	1e-85/2e-87	0/0		1e-93/8e-93	3e-88/3e-95
Strongylocentrotus purpuratus	XP_785258.2	XP_798164.3	XP_784617.1	XP_003728406.1	XP_788012.1	XP_788784.3	XP_782975.2	XP_792188.3/-**A**<br>XP_001182209.2-**B**	XP_799003.3
	5e-98/5e-102	0/0	4e-102/3e-101	3e-146/1e-146	3e-87/1e-85	0/0	1e-74/5e-81	6e-87/6e-86<br>6e-72/4e-71	6e-82/3e-83
Aedes aegypti	XP_001652185.1	XP_001657405.1	XP_001653814.1	XP_001648501.1	XP_001656034.1	XP_001658127.1	XP_001662677.1	XP_001651349.1	-
	7e-91/1e-89	0/0	2e-92/2e-91	3e-123/9e-121	2e-81/2e-81	0/0	1e-40/1e-39	5e-80/7e-79	-
Anopheles gambiae	XP_001688657.1	XP_307895.3	XP_321189.4	XP_321109.5	XP_318369.5	XP_315888.4	XP_317017.4/**A**<br>XP_318745.2/**B**	XP_563593.1	XP_314090.4
	2e-89/5e-91	0/0	6e-94/6e-93	2e-117/4e-122	9e-81/3e-79	0/0	1e-37/9e-37<br>3e-13/3e-12	7e-81/5e-83	3e-74/3e-73
Drosophila melanogaster	NP_648378.1	NP_573384.1	NP_001259714.1/**A**<br>NP_723798.1/**B**	NP_650858.1	NP_569903.2/**A**<br>NP_728606.1/**B**	NP_001014546.1	NP_651715.1	NP_524779.1	NP_648353.3

	6e-91/1e-89	0/0	3e-90/9e-41	7e-134/2e-137	6e-72/9e-70<br>1e-53/7e-52	1e-171/3e-169	1e-34/4e-31	2e-90/7e-90	2e-77/1e-74
Tribolium castaneum	XP_001806886.1	XP_975602.1	XP_001816527.1	XP_001814215.1	XP_975323.1	XP_967144.2	XP_967118.1		XP_971827.1
	2e-97/4e-99	0/0	3e-98/3e-97	3e-161/8e-162	8e-85/6e-83	0/0	3e-72/2e-71		6e-80/4e-79
Daphnia pulex	EFX89571.1	EFX89563.1	EFX65578.1	EFX68033.1	EFX80598.1	EFX84706.1	EFX69334.1	EFX72781.1	EFX79812.1
	1e-100/3e-99	0/0	1e-99/1e-98	1e-150/1e-166	5e-82/7e-81	0/0	8e-66/2e-63	2e-86/1e-85	7e-79/3e-75
Caenorhabditis elegans	NP_501392.1	NP_497180.3	NP_505053.1	NP_498494.2	NP_504411.1	NP_505179.1	NP_504573.1	NP_508553.1-**A**<br>NP_498219.1-**B**	NP_001122468.1
	4e-91/1e-88	0/0	2e-74/1e-73	7e-54/4e-53	7e-59/4e-57	4e-36/1e-34	2e-20/1e-19	7e-51/4e-50<br>2e-20/1e-19	2e-33/3e-30
Nematostella vectensis	XP_001628317.1	XP_001632534.1	XP_001641593.1	XP_001634156.1	XP_001640663.1	XP_001623260.1	XP_001624471.1	XP_001624038.1	XP_001625565.1
	2e-100/4e-99	0/0	1e-101/1e-100	0.075/0.026	8e-81/5e-79	4e-49/6e-51	1e-70/9e-70	2e-90/2e-89	1e-76/5e-81
Trichoplax adhaerens	XP_002111411.1	XP_002112930.1	XP_002117256.1	XP_002108571.1	XP_002108356.1	XP_002116358.1	XP_002113522.1	XP_002111655.1	XP_002109233.1
	1e-88/4e-88	0/0	4e-84/6e-83	2e-117/3e-116	2e-78/1e-80	6e-142/3e-142	8e-54/8e-53	1e-78/2e-77	4e-44/6e-43
Monosiga brevicollis	XP_001748698.1	XP_001742554.1	XP_001745212.1	XP_001748603.1	XP_001749824.1	XP_001749781.1	XP_001750492.1	XP_001742594.1	
	7e-79/2e-77	4e-142/1e-148	2e-74/3e-73	2e-52/6e-51	2e-53/6e-53s	6e-13/1e-24	7e-11/8e-15	1e-56/2e-55	
**FUNGI**									
Neurospora crassa	XP_960811.1	XP_962452.1	XP_960754.1	XP_959631.1	XP_959599.1	XP_956120.2	XP_959627.1	XP_964737.1	
	2e-50/6e-50	6e-93/6e-96	2e-35/7e-35	0.001/5e-06	1e-44/5e-44	4e-15/7e-15	2e-04/7e-04	7E-16/3E-14	
Fusarium graminearum	XP_390475.1	XP_382950.1	XP_382082.1	XP_385945.1	XP_386552.1	XP_380485.1	XP_388962.1	XP_388951.1	
	6e-48/1e-47	2e-93/5e-90	2e-38/8e-38	1e-04/0.027	6e-47/2e-46	4e-25/1e-26		9e-14/3e-12	

Aspergillus niger	XP_001392237.2	XP_001389144.1	XP_001388868.1	XP_001401551.2	XP_001397675.1	XP_001395566.2	XP_001401634.1	XP_001395917.1	
	8e-54/3e-54	2e-106/3e-102	6e-40/9e-40	2e-08/3e-06	4e-48/4e-48	3e-21/5e-21	7e-12/1e-11	1E07/1E-06	
Saccharomyces cerevisiae	NP_011877.1	NP_010244.1	NP_015456.2	NP_014566.1	NP_015376.1	NP_010861.3	NP_013785.1	NP_014896.3	
	7e-51/2e-50	2e-93/7e-95	1e-45/5e-45	3e-05/2e-06	3e-43/3e-43	6e-09/3e-08	0.035/0.17	5e-07/2e-06	
Kluyveromyces lactis	XP_454097.1	XP_455464.1	XP_452031.1	XP_454752.1	XP_452068.1	XP_451085.1	XP_454790.1	XP_452637.1	
	3e-111/3e-110	0/0	7e-96/9e-98	5e-130/1e-124	1e-86/1e-85	8e-175/7e-174	4e-06/2e-05	1e-35/7e-35	
Candida albicans	XP_719378.1	XP_714733.1	XP_718835.1	XP_719033.1	XP_722835.1	XP_721703.1	XP_722734.1	XP_717442.1	
	4e-87/1e-86	0/0	7e-62/2e-61	4e-20/3e-18	2e-57/7e-57	4e-34/2e-39	8e-20/1e-17	4e-17/1e-16	
Schizosaccharomyces pombe	NP_594309.1	NP_588160.1<br>NP_596495.1	NP_587922.1	NP_595632.1	NP_596246.1	NP_596720.1	NP_588054.1	NP_588274.1	
	9e-56/5e-55	2e-104/2e-104<br>1E-36/7E-37	3e-55/2e-52	6e-07/2e-08	7e-50/5e-49	3e-11/3e-11	9e-12/5e-11	7e-11/5e-10	
Pneumocystis murina	EMR09126.1	EMR08274.1	EMR08470.1	EMR10944.1	EMR08451.1	EMR09747.1	EMR08653.1	EMR11455.1	
	3e-50/3e-49	7e-98/2e-96	2e-48/3e-47	4e-14/2e-14	2e-33/1e-32	6e-28/7e-27	1e-08/1e-07	6e-43/2e-41	
Coprinopsis cinerea	XP_001837459.1	XP_002910266.1	XP_001832867.2	XP_001833237.2	XP_001834267.2	XP_001839874.1	XP_001828773.2	XP_001830348.1	
	2e-56/5e-56	8e-77/1e-76	1e-34/3e-34	2e-67/2e-66	9e-43/3e-43	1e-07/2e-08	6e-18/5e-17	1e-10/3e-10	
Cryptococcus gattii	XP_003194497.1	XP_003194436.1	XP_003195355.1	XP_003193706.1	XP_003193896.1	XP_003194296.1	XP_003193757.1	XP_003195679.1	
	4e-55/2e-54	5e-75/5e-75	1e-33/9e-31	0.065/1e-04	6e-27/5e-26	6e-16/1e-14	7e-15/1e-13	1e-06/8e-06	
Ustilago maydis	XP_757531.1	XP_756726.1	XP_760424.1	XP_762208.1	XP_757770.1	XP_756983.1	XP_756426.1	XP_757178.1	
	1e-35/4e-35	1e-85/6e-84	3e-42/2e-41	6e-08/2e-05	3e-45/1e-44	1e-04/8e-04	3e-23/2e-22	2e-51/8e-49	

Rhizopus delemar	EIE76483.1	EIE91923.1	EIE79187.1	EIE90995.1		EIE87740.1-A<br>EIE87861.1-B	EIE87147.1	EIE92156.1-AY<br>EIE89248.1-BH	
	1e-52/4e-52	2e-97/9e-97	3e-35/8e-35	3e-73/4e-72		4e-19/3e-18<br>2e-17/1e-16	4e-05/1e-07	7e-06/2e-05/<br>5E-51/7E-50	
Batrachochytrium dendrobatidis	EGF78314.1	EGF82906.1	EGF83148.1	EGF81799.1	EGF83881.1	EGF78302.1		EGF78648.1- AH<br>EGF78926.1-BH	
	3e-56/9e-56	4e-78/1e-76	2e-37/1e-36	9e-83/2e-81	4e-45/2e-44	1e-41/4e-37		1e-31/6e-34<br>1e-10/3e-09	
**MICROSPORIDIA**									
Vittaforma corneae	ELA41368.1	-	ELA41574.1	-	-	-	-	ELA41057.1	-
	7e-06/1e-04		7e-24/2e-22					2e-21/2e-19	
Enterocytozoon bieneusi	XP_002649714.1	-	XP_002649725.1	-	-	-	-	-	-
	1e-08/3e-06		3e-06/6e-05						
Encephalitozoon cuniculi	NP_585802.1	-	NP_586095.1	-	-	-	-	NP_585856.1	-
	2e-08/3e-12		4e-27/5e-26					6e-04/5e-19	
Encephalitozoon intestinalis	XP_003073006.1	-	XP_003073284.1	-	-	-	-	XP_003073060.1	-
	2e-08/4e-07		3e-26/9e-25					9e-75/2e-74	
Encephalitozoon hellem	XP_003887395.1	-	XP_003887669.1	-	-	-	-	-	-
	1e-08/3e-07		3e-28/8e-27						
Nosema ceranae	XP_002995490.1	-	XP_002994996.1	-	-	-	-	XP_002996047.1	-
	2e-08/8e-12		7e-21/2e-24					1e-24/2e-22	
Nosema bombycis	-	-	EOB13495.1	-	-	-	-	EOB12821.1	-
			4e-25/5e-32					1e-04/5e-05	

Vavraia culicis	ELA46817.1	-	ELA47084.1	4-	-	-	-	ELA47635.1	-
	6e-10/1e-08		2e-23/2e-26					7e-21/6e-19	
Trachipleistophora hominis	ELQ76313.1	-	ELQ74754.1	-	-	-	-	ELQ76577.1	-
	6e-30/2e-30		2e-23/2e-26					1e-13/6e-12	
Nosema parisii	EIJ88594.1	-	EIJ89578.1	-	-	-	-	EIJ87901.1	-
	3e-20/1e-21		4e-12/1e-14					7e-19/5e-17	
**AMOEBOZOA**									
Dictyostelium discoideum	XP_643534.2	XP_643789.1	XP_643184.2	XP_629232.1	XP_638028.1	XP_646863.1		XP_002649166.1	XP_636154.1
	2e-58/2e-58	2e-109/9e-106	8e-78/8e-77	5e-67/7e-67	2e-50/2e-50	9e-13/5e-16		5e-48/5e-50	1e-07/2e-04
Entamoeba histolytica	XP_652840.1/<br>XP_649513.1	XP_648732.1/<br>XP_650321.2	XP_654064.2	XP_649927.1	XP_650166.1	XP_655063.1	XP_001914091.1	XP_651314.1	XP_649081.1
	8e-56/2e-53<br>3e-43/3e-44	6e-40/1e-41<br>5e-33/4e-34	3e-64/8e-61	0.004/0.046	4e-41/1e-38	7e-14/8e-11	2e-12/2e-14	2e-09/3e-08	3e-10/3e-09
**PLANTS**									
Chlorella variabilis	XP_005850279.1	XP_005847916.1	XP_005846769.1	XP_005848802.1	XP_005846533.1	XP_005845397.1	XP_005846799.1	XP_005850764.1	XP_005851588.1
	8e-08/1e-77	2e-166/7e-172	2e-72/4e-71	5e-16/2e-24	1e-54/1e-53	2e-07/4e-13	3e-41/1e-40	2e-46/4e-45	3e-13/5e-12
Volvox carteri	XP_002949928.1	XP_002946235.1	XP_002950281.1	XP_002959409.1	XP_002952437.1	XP_002951162.1	XP_002950156.1	XP_002950586.1	XP_002949307.1
	3e-68/5e-69	2e-173/4e-180	6e-80/6e-79	0.29/5e-10	5e-60/7e-60	1e-07/1e-18	5e-22/1e-20	2e-42/2e-41	2e-08/1e-07
Minuartia pusilla	XP_003062110.1	XP_003062812.1	XP_003057625.1	XP_003058109.1	XP_003063612.1	XP_003055409.1	XP_003058022.1-A<br>XP_003057542.1-B<br>XP_003054998.1-C	XP_003058818.1	XP_003057553.1
	7e-65/2e-63	2e-141/1e-152	1e-70/3e-69	4e-46/6e-47	2e-46/1e-45	6e-17/2e-16	2e-26/3e-25<br>3e-24/2e-25<br>5E-12/2E-10	2e-30/2e-29	2e-07/4e-06

Ostreococcus lucimarinus	XP_001418332.1	XP_001415662.1	XP_001417209.1	XP_001420128.1	XP_001422608.1	XP_001420852.1	XP_001417476.1	XP_001421022.1	XP_001416376.1
	6e-56/3e-57	5e-123/3e-124	1e-65/4e-64	1e-24/1e-31	3e-49/2e-48	5e-34/6e-39	9e-28/2e-26	2e-36/5e-35	5e-83/5e-83
Ostreococcus tauri	XP_003080092.1	XP_003074639.1	XP_003078698.1	XP_003081593.1	XP_003083105.1	XP_003082419.1	XP_003078705.1	XP_003081403.1-A<br>XP_003082790.1-B	XP_003074795.1
	7e-51/1e-49	2e-110/9e-115	2e-62/6e-61	5e-17/4e-18	6e-51/1e-49	4e-21/1e-25	1e-27/2e-26	1e-44/2e-43<br>3e-10/1e-08	5e-06/6e-05
Physcomitrella patens	XP_001781748.1/<br>XP_001755278.1	XP_001783197.1/<br>XP_001764313.1/<br>XP_001776114.1	XP_001781047.1	XP_001784815.1	XP_001782364.1	XP_001777179.1	XP_001757751.1-A<br>XP_001767987.1-B<br>XP_001768065.1-C	XP_001762002.1	XP_001766566.1-A<br>XP_001755869.1-B
	1e-73/1e-74<br>5e-42/5e-42	0/0<br>0/0<br>0/0	3e-72/2e-71	1e-44/4e-44	6e-56/2e-55	3e-64/4e-68	3e-32/1e-34<br>1e-36/1e-35<br>9E-29/4E-29	1e-58/9e-58	3e-25/7e-22<br>3e-21/7e-18
Oryza sativa	NP_001054011.1	NP_001043582.1	NP_001050988.1	NP_001055256.1	NP_001067995.1	BAD36037.1	NP_001055426.1-A<br>NP_001048264.1-B	NP_001043544.2	NP_001173121.1
	2e-71/2e-70	0/0	8e-72/1e-71	6e-49/1e-48	7e-52/2e-51	1e-60/3e-61	4e-27/4e-27<br>9e-23/1e-25	1e-09/2e-06	1e-10/2e-16
Zea mays	NP_001141256.1/<br>NP_001140477.1/	NP_001142479.1	NP_001105069.1	XP_008650616.1	NP_001150590.1	XP_008679211.1	NP_001149697.1	NP_001148868.1	NP_001131664.1
	4e-133/9e-136<br>1e-72/5e-73	0/0	6e-73/2e-72	9e-22/2e-26	4e-53/2e-53	4e-17/4e-14	4e-24/2e-20	5e-50/2e-49	3e-20/1e-19
Vitis vinifera	XP_002273592.1	XP_002284882.1	XP_002278246.1	XP_002273069.1	XP_002276364.1	XP_002282486.2	XP_002263749.2	XP_002284766.1	XP_002273780.1
	4e-70/2e-69	0/0	8e-72/2e-71	1e-49/3e-52	3e-55/2e-54	1e-22/2e-22	1e-29/6e-29	2e-57/4e-54	1e-22/9e-26
Arabidopsis thaliana	NP_196882.1	NP_178157.2	NP_563677.1	NP_200653.2	NP_181348.1	NP_178872.2	NP_683313.2	NP_196695.1	NP_974793.1/<br>NP_186948.1
	2e-69/8e-69	0/0	7e-73/2e-72	4e-45/8e-51	3e-54/8e-54	1e-61/2e-57	5e-31/2e-33	3e-53/1e-52	9e-18/3e-17<br>2e-14/6e-14
Populus trichocarpa	XP_002314058.1/<br>XP_002298415.1	XP_002299630.1/<br>XP_002304180.1	XP_002307586.1-A<br>XP_002300841.1-B	XP_002319956.1	XP_002317002.1-A<br>XP_002298931.1-B	XP_002308056.1	XP_002318313.1	XP_002324274.1-A<br>XP_002308640.1-B	XP_002319255.1-A<br>XP_002325388.1-B
	1e-70/4e-69<br>2e-69/3e-68	7e-167/2e-167<br>8e-81/1e-85	9e-72/5e-71<br>4e-56/2e-53	6e-43/4e-42	6e-55/7e-54<br>5e-54/5e-53	5e-64/1e-64	5e-32/2e-34	3e-53/2e-52<br>2e-52/1e-51	3e-20/2e-19<br>1e-19/6e-19
Ricinus communis	XP_002517754.1	XP_002517521.1	XP_002520731.1	XP_002516347.1	XP_002512831.1	XP_002531946.1	XP_002533736.1	XP_002514360.1	XP_002520055.1
	1e-71/6e-70	0/0	1e-73/1e-72	1e-45/1e-47	7e-55/1e-53	3e-64/1e-60	1e-35/1e-34	7e-59/2e-55	4e-21/3e-20

**EXCAVATS**									
Trypanosoma cruzi	XP_817467.1	XP_807954.1	XP_808666.1	XP_810210.1	XP_804793.1	EKF28593.1	XP_813517.1	XP_812778.1	
	3e-58/2e-57	1e-73/7e-81	5e-53/2e-52	1e-17/2e-18	2e-30/1e-32	8e-07/7e-06	0.024/0.010	1e-25/5e-25	
Leishmania donovani	XP_003859238.1	XP_003865255.1	XP_003861333.1	XP_003862768.1	XP_003860783.1	XP_003859962.1	XP_003861138.1	XP_003857978.1	
	4e-54/2e-55	1e-72/5e-77	3e-55/2e-53	6e-21/9e-20	3e-36/1e-34	6e-06/6e-06	0.17/1,2	9e-23/2e-21	
Leishmania major	XP_001681770.1	XP_001686706.1	XP_001683754.1	XP_001684642.1	XP_001683197.1	XP_001682355.1	XP_001681528.1	XP_003721691.1	XP_001682540.1
	3e-54/1e-54	4e-69/7e-76	4e-52/1e-53	6e-22/8e-21	2e-35/9e-34	8e-04/1e-05	0.42/0.10	4e-22/8e-21	0.002/0.21
Naegleria gruberi	XP_002678584.1	XP_002679243.1	XP_002683334.1	XP_002680435.1	XP_002674973.1	XP_002680625.1	XP_002679876.1	XP_002677269.1	XP_002673458.1
	1e-65/6e-67	5e-106/7e-109	5e-76/6e-75	4e-42/4e-41	4e-05/8e-05	3e-39/1e-37	0.045/0.051	7e-33/8e-32	1e-09/2e-08
Giardia lamblia	XP_001708371.1	XP_001704054.1	XP_001705505.1		XP_001708463.1		EFD95470.1		
	7e-38/8e-37	6E-04/8E-04	7e-31/6e-30		2e-25/7e-25		3E-04/5E-04		
Trichomonas vaginalis	XP_001320341.1/<br>XP_001321406.1/<br>XP_001319170.1/<br>XP_001328034.1	XP_001318906.1/<br>XP_001581881.1/<br>XP_001317430.1	XP_001582947.1	XP_001319977.1	XP_001320347.1	XP_001330429.1	XP_001579481.1-A<br>XP_001310507.1-B<br>XP_001304312.1-C<br>XP_001314637.1-D<br>XP_001330572.1-E	XP_001321375.1-A<br>XP_001330697.1-B<br>XP_001584123.1-C<br>XP_001329893.1-D<br>XP_001307166.1-E	XP_001300699.1
	8e-37/3e-36<br>1e-35/1e-35<br>6e-35/2e-37<br>1e-33/8e-33	1e-23/3e-28<br>2e-21/1e-25<br>8e-18/2e-17	7e-51/3e-50	0.091/0.21	2e-37/1e-37	0.021/0.090	4e-38/5e-40<br>1e-29/5e-29<br>1e-27/7e-27<br>7e-19/1e-19<br>3e-11/9e-11	4e-24/2e-23<br>5e-24/2e-23<br>1e-09/5e-09<br>7e-09/3e-08<br>8e-09/4e-08	6e-08/2e-07
**CHROMALVEOLATA**									
Perkinsus marinus	XP_002785590.1	XP_002773906.1	XP_002767399.1	XP_002767529.1	XP_002785517.1	XP_002774903.1	XP_002767502.1	XP_002778867.1	XP_002776798.1-A<br>XP_002765203.1-B
	4e-64/6e-63	1e-57/9e-62	9e-53/9e-55	9e-15/8e-14	2e-43/4e-42	6e-20/7e-20	1e-24/1e-23	4e-26/8e-25	5e-17/9e-18<br>7E-04/0.007

Toxoplasma gondii	XP_002370728.1	XP_002366057.1	XP_002368415.1	XP_002367657.1	XP_002365121.1		XP_002367927.1	XP_002365184.1	XP_002365228.1
	4e-54/5e-53	3e-61/1e-58	2e-58/8e-58	9e-06/5e-05	9e-11/3e-10		4e-17/3e-16	2e-32/1e-32	2e-11/1e-07
Plasmodium falciparum	XP_001347321.1	XP_001350828.1	XP_001351025.1		XP_002808814.1		XP_001349973.1		XP_001349433.2
	1e-35/8e-44	7e-25/2e-23	9e-38/5e-40		1e-19/6e-19		4e-11/4e-10		6e-06/1e-05
Paramecium tetraurelia	XP_001457344.1	XP_001433619.1/<br>XP_001436823.1	XP_001444692.1	XP_001446304.1	XP_001431642.1-A<br>XP_001447095.1-B	XP_001425968.1		XP_001455694.1-A<br>XP_001425966.1-B<br>XP_001442627.1-C<br>XP_001454240.1-D	XP_001462395.1
	7e-57/4e-54	1e-87/4e-86<br>1e-78/2e-73	3e-59/1e-58	6e-08/2e-07	3e-44/4e-44<br>6e-44/1e-43	4e-17/6e-15		1e-38/6e-38<br>3e-38/7e-38<br>2e-15/4e-15<br>3e-13/3e-12	4E-07/1E-07
Phaeodactylum tricornutum	XP_002176567.1	XP_002181258.1	XP_002181755.	XP_002182373.1	XP_002176589.1	XP_002184196.1	XP_002178919.1	XP_002179446.1	XP_002186492.1
	3e-67/6e-66	1e-133/3e-134	4e-71/7e-70	3e-23/2e-21	9e-44/5e-43	8e-20/5e-15	7e-24/1e-22	1e-34/2e-33	5e-23/5e-25

Supplementary Table 2

Position 2

	NATs	D. discoideum-reviev	D. discoideur	E. histolytica·	P. tetraurelia·	P. marinus-ur	P. tricornutur	P. falciparum
iMet-Cys	**A**	0.1388	0.5196	0.2079	0.2843	0.7166	0.497	0.6803
iMet-Ala	**A,(E),(F)**	1.6012	1.5231	1.3265	0.958	1.4643	1.1633	1.8771
iMet-Gly	**A,(F)**	0.9705	0.9586	0.8436	1.5492	0.7811	0.6153	1.274
iMet-Ser	**A,(E),(F)**	1.7986	1.4454	3.0223	1.7972	2.3545	1.6924	1.4403
iMet-Thr	**A,(E),(F)**	1.6282	1.001	1.4428	0.5239	1.3287	1.3939	0.7583
iMet-Val	**A,(C), (E),(F)**	0.8828	0.9932	0.4485	0.3257	1.0331	0.7507	1.0334
iMet-Asp	**B**	1.2453	1.5839	0.7651	1.2093	0.7055	0.6759	0.9493
iMet-Glu	**B**	1.4922	1.4325	1.2929	0.6801	0.4757	0.7438	1.1314
iMet-Asn	**B**	0.9637	1.1328	1.5318	1.9128	0.7585	1.3413	0.9295
iMet-Gln	**B,(F)**	0.3687	0.4081	1.1241	1.1627	0.6863	0.7633	0.5913
iMet-His	**C**	0.1446	0.396	0.3412	0.747	0.6107	0.5578	0.5738
iMet-Met	**C,E,F**	0.8392	0.6855	0.3303	0.4375	0.5319	0.8527	1.0954
iMet-Tyr	**C,E,F**	0.4389	0.6548	0.2932	0.6362	0.5238	0.4946	0.7243
iMet-Lys	**C,E**	1.2706	1.5673	1.0848	1.2544	0.6725	2.7452	1.242
iMet-Phe	**C,E**	0.7854	0.7329	0.7997	0.8882	1.0629	0.5256	1.0088
iMet-Ile	**C,F**	0.8677	0.8831	0.5228	0.9392	0.6023	0.8546	0.7928
iMet-Leu	**C,F**	0.4555	0.502	0.501	0.715	0.8556	0.5587	0.7745
iMet-Arg		0.7109	0.6114	0.5877	0.8118	0.7579	1.0878	1.3426
iMet-Pro		0.3913	0.3713	0.5413	0.457	1.5674	1.1324	0.732
iMet-Trp		0.2778	0.3432	0.3003	0.3315	0.5619	0.5799	1.0745

Position 3

	NATs	D. discoideum-reviev	D. discoideur	E. histolytica·	P. tetraurelia·	P. marinus-ur	P. tricornutur	P. falciparum
iMet-X-Cys	**A**	0.4336	0.6276	0.8102	0.8803	0.934	0.6272	0.8505
iMet-X-Ala	**A,(E),(F)**	0.6288	0.6436	1.0275	0.7304	1.0013	0.7885	0.6851
iMet-X-Gly	**A,(F)**	0.6084	0.6837	0.8335	0.5832	0.8593	0.5697	0.9141

iMet-X-Ser	**A,(E),(F)**	1.2508	1.1243	1.1801	1.3836	1.7575	1.2951	1.0705
iMet-X-Thr	**A,(E),(F)**	1.2453	1.1394	1.2433	1.0778	1.3312	1.335	1.0774
iMet-X-Val	**A,(C), (E),(F)**	0.6991	0.766	0.9041	0.6255	0.8846	0.7141	1.2527
iMet-X-Asp	**B**	0.9259	0.9457	1.0282	0.8263	0.9243	0.8196	0.9111
iMet-X-Glu	**B**	1.2111	1.0291	1.2178	0.8085	0.7756	0.7557	1.0428
iMet-X-Asn	**B**	0.9973	1.0932	1.0679	1.4866	1.3329	1.3805	0.8533
iMet-X-Gln	**B,(F)**	0.6908	0.7736	1.1338	1.4645	0.7604	0.9954	0.6694
iMet-X-His	**C**	0.9199	0.8218	0.9184	0.9498	0.9488	0.9386	0.8166
iMet-X-Met	**C,E,F**	0.4402	0.6061	0.4883	0.4626	0.5953	0.6595	0.7696
iMet-X-Tyr	**C,E,F**	0.7388	0.8635	0.6742	0.9156	0.8706	0.9474	0.7974
iMet-X-Lys	**C,E**	1.4568	1.5046	1.0878	1.192	0.7927	1.7871	1.1722
iMet-X-Phe	**C,E**	1.082	1.0773	1.0154	0.8969	1.1135	1.1678	1.1473
iMet-X-Ile	**C,F**	1.3137	1.0953	0.8901	0.8846	1.1328	1.0231	1.0852
iMet-X-Leu	**C,F**	0.88	0.8973	0.8394	0.7163	0.7919	0.8788	0.9828
iMet-X-Arg		1.4457	1.4414	1.4859	1.1904	0.9476	1.3418	1.7465
iMet-X-Pro		0.5781	0.6447	0.6438	0.9236	1.0355	1.0063	0.8866
iMet-X-Trp		0.5247	0.5249	0.8521	0.4693	0.5954	0.7564	0.5931

Position 4

	**NATs**	D. discoideum-reviev	D. discoideur	E. histolytica·	P. tetraurelia·	P. marinus-ur	P. tricornutur	P. falciparum
iMet-X-X-Cys	**A**	0.3642	0.5871	0.5318	1.313	0.8486	0.7961	0.9771
iMet-X-X-Ala	**A,(E),(F)**	0.6742	0.6467	0.7476	0.8138	0.9599	0.8427	0.7785
iMet-X-X-Gly	**A,(F)**	0.5456	0.5429	0.6206	0.6563	0.7721	0.5332	0.9352
iMet-X-X-Ser	**A,(E),(F)**	0.8756	0.8504	1.1183	1.1618	1.526	1.4179	1.0506
iMet-X-X-Thr	**A,(E),(F)**	0.9471	0.9301	1.4133	0.9564	1.2656	1.284	1.0094
iMet-X-X-Val	**A,(C), (E),(F)**	0.8012	0.8785	0.9821	0.8213	0.8214	0.7913	1.227
iMet-X-X-Asp	**B**	0.7824	0.7226	0.7509	0.7661	0.7834	0.6237	0.5894
iMet-X-X-Glu	**B**	0.8805	0.8311	1.0298	0.8699	0.7348	0.6765	0.7852
iMet-X-X-Asn	**B**	1.1147	1.2312	0.9011	1.1662	1.0939	1.1098	0.6719

iMet-X-X-Gln	**B,(F)**	0.7748	0.8017	1.3125	1.1991	0.9938	1.0036	0.9817
iMet-X-X-His	**C**	0.9593	0.7866	0.8798	1.0755	0.9488	0.9533	1.3973
iMet-X-X-Met	**C,E,F**	0.6328	0.5109	0.4575	0.4292	0.5692	0.5859	0.7228
iMet-X-X-Tyr	**C,E,F**	0.9362	0.9659	0.6714	0.9542	0.8356	0.8592	1.1062
iMet-X-X-Lys	**C,E**	1.4045	1.3348	0.8995	1.0411	0.9837	1.2413	1.0373
iMet-X-X-Phe	**C,E**	1.5269	1.2299	1.2282	1.1025	1.1859	1.1256	1.4517
iMet-X-X-Ile	**C,F**	1.3805	1.3541	1.0679	1.0631	1.0984	1.313	1.1746
iMet-X-X-Leu	**C,F**	1.2118	1.306	1.1291	0.988	0.9907	1.1358	1.2969
iMet-X-X-Arg		1.1103	1.0025	1.1853	1.1519	1.0542	1.2371	1.3813
iMet-X-X-Pro		0.9227	0.8177	1.2515	1.0021	1.3321	1.1649	1.0034
iMet-X-X-Trp		0.2778	0.498	1.0408	0.6368	0.6029	0.827	0.9792

Position 5

	**NATs**	D. discoideum-reviev	D. discoideur	E. histolytica·	P. tetraurelia·	P. marinus-ur	P. tricornutur	P. falciparum
iMet-X-X-X-Cys	**A**	0.555	0.6175	0.6654	1.2718	0.9573	0.9987	0.9185
iMet-X-X-X-Ala	**A,(E),(F)**	0.7974	0.6936	0.6703	0.8023	1.0018	0.8354	0.6495
iMet-X-X-X-Gly	**A,(F)**	0.5939	0.5826	0.7205	0.6282	0.8641	0.6305	0.9637
iMet-X-X-X-Ser	**A,(E),(F)**	0.9931	0.8581	1.027	1.1473	1.4304	1.2625	1.0784
iMet-X-X-X-Thr	**A,(E),(F)**	0.8786	0.9259	1.2253	1.0542	1.2962	1.2433	0.8461
iMet-X-X-X-Val	**A,(C), (E),(F)**	0.842	0.8317	1.0043	0.8657	0.9645	0.7923	1.1062
iMet-X-X-X-Asp	**B**	0.8194	0.8445	0.8681	0.8304	0.8104	0.7257	0.6093
iMet-X-X-X-Glu	**B**	1.0871	0.9289	0.9666	0.8723	0.8034	0.7318	0.7695
iMet-X-X-X-Asn	**B**	1.1387	1.2691	0.9373	1.011	1.1042	1.0503	0.837
iMet-X-X-X-Gln	**B,(F)**	0.9008	0.808	1.4705	1.1954	0.9742	1.0755	0.8431
iMet-X-X-X-His	**C**	0.7097	0.8245	1.0179	0.9899	0.8986	0.8973	0.736
iMet-X-X-X-Met	**C,E,F**	0.3302	0.4887	0.5157	0.5165	0.586	0.5184	0.7558
iMet-X-X-X-Tyr	**C,E,F**	0.9289	1.0256	0.9119	0.9619	0.832	0.8615	1.2685
iMet-X-X-X-Lys	**C,E**	1.5613	1.38	1.0635	0.9728	0.9448	1.4848	1.2866
iMet-X-X-X-Phe	**C,E**	1.2578	1.2341	1.0315	1.0893	0.9776	1.1796	1.4513

iMet-X-X-X-Ile	**C,F**	1.3532	1.342	1.03	1.102	1.0858	1.3627	1.0834
iMet-X-X-X-Leu	**C,F**	1.0403	1.1196	1.1255	1.0571	0.9891	1.1095	1.0988
iMet-X-X-X-Arg		0.9585	0.9188	1.0212	1.0777	0.964	1.1572	1.3358
iMet-X-X-X-Pro		0.6365	0.6604	1.146	1.122	1.194	0.9561	0.7926
iMet-X-X-X-Trp		0.5247	0.6729	0.6218	0.5678	0.6364	0.8219	1.0769

Position 6

	**NATs**	D. discoideum-reviev	D. discoideur	E. histolytica·	P. tetraurelia·	P. marinus-ur	P. tricornutur	P. falciparum
iMet-X-X-X-X-Cys	**A**	0.6071	0.6276	0.7927	0.9669	0.8253	0.8395	1.166
iMet-X-X-X-X-Ala	**A,(E),(F)**	0.7974	0.7077	0.9364	0.9036	1.0228	0.8654	0.9816
iMet-X-X-X-X-Gly	**A,(F)**	0.5891	0.5917	0.7111	0.7575	0.8858	0.6721	0.9007
iMet-X-X-X-X-Ser	**A,(E),(F)**	1.2183	0.9956	1.0669	1.2807	1.4646	1.2413	1.1562
iMet-X-X-X-X-Thr	**A,(E),(F)**	0.9471	0.9082	1.1756	1.0106	1.1991	1.1595	1.057
iMet-X-X-X-X-Val	**A,(C), (E),(F)**	1.0104	0.9598	1.0279	0.9288	0.9699	0.839	1.2145
iMet-X-X-X-X-Asp	**B**	0.9212	0.8632	0.9068	0.8174	0.8669	0.7443	0.672
iMet-X-X-X-X-Glu	**B**	0.8639	0.8653	0.8468	0.913	0.861	0.7318	0.8594
iMet-X-X-X-X-Asn	**B**	1.0572	1.2119	0.9386	0.9483	0.9677	1.023	0.7717
iMet-X-X-X-X-Gln	**B,(F)**	0.7982	0.7715	1.1599	1.1008	0.9784	0.9137	0.7669
iMet-X-X-X-X-His	**C**	0.6702	0.7974	1.2124	0.9413	0.9728	1.0065	0.7073
iMet-X-X-X-X-Met	**C,E,F**	0.4677	0.4697	0.3708	0.5165	0.5374	0.546	0.7459
iMet-X-X-X-X-Tyr	**C,E,F**	0.8631	0.962	0.8377	0.8696	0.8373	0.8546	1.0499
iMet-X-X-X-X-Lys	**C,E**	1.4535	1.3147	0.9632	1.0315	0.9138	1.2926	1.0656
iMet-X-X-X-X-Phe	**C,E**	1.0601	1.1958	1.0641	1.0295	1.0195	1.325	1.5677
iMet-X-X-X-X-Ile	**C,F**	1.25	1.3349	1.0516	1.0441	0.9537	1.2288	1.0652
iMet-X-X-X-X-Leu	**C,F**	1.0909	1.0855	1.1641	1.0708	0.9558	1.2224	1.1473
iMet-X-X-X-X-Arg		0.9745	1.0725	1.183	1.0887	1.0408	1.1936	1.4404
iMet-X-X-X-X-Pro		0.8	0.6967	1.2208	1.2367	1.2698	0.9995	0.8532
iMet-X-X-X-X-Trp		0.6173	0.6258	1.0271	0.6466	0.5656	0.7362	1.0164

T. gondii-unr	G. lamblia-ur	L. donovanii-	N. gruberi-ur	T. vaginalis-u	A. niger-unre	B. dendrobat	C. albicans-re	C. albicans-u	S. cerevisiae-	S. cerevisiae-
0.693	0.6702	0.5857	0.469	0.364	0.4586	0.5201	0.5446	0.4844	0.6036	0.5989
1.7932	1.1385	0.8468	1.2852	0.8129	2.3015	1.4048	1.6349	1.5695	1.5638	1.4294
0.8588	0.8337	0.7907	0.9234	0.78	0.8458	0.7728	0.6208	0.7562	0.976	0.9339
0.9391	1.6152	2.117	2.2782	2.9769	2.0178	1.5665	2.8482	3.0743	2.5526	2.659
0.9545	1.0402	0.9065	1.028	1.6036	1.243	1.4806	1.1801	1.4882	1.2124	1.3389
0.722	0.5768	0.4013	0.682	0.5457	0.6799	0.7249	0.6788	0.8555	1.046	1.0659
0.9792	1.0571	0.7988	0.6055	0.9142	0.9968	1.0935	0.5465	0.6428	0.8471	0.9053
1.2052	0.907	0.8693	0.5853	0.7603	0.8064	0.7273	0.316	0.4946	0.777	0.6605
0.966	1.0268	1.1371	1.0347	0.9836	0.6836	1.2072	0.7116	0.7518	0.8619	0.8828
0.9401	1.2127	1.0785	1.077	0.9432	0.6048	0.7376	0.4568	0.4089	0.6208	0.6567
1.1432	1.1462	0.8888	0.7151	0.5636	0.6543	0.9437	0.5804	0.3854	0.6276	0.5662
1.2375	0.5198	0.807	0.8402	0.3364	0.3876	0.3741	0.5847	0.546	0.7736	0.6204
1.0534	0.5355	0.5835	0.5829	0.7987	0.4795	0.8326	0.3783	0.5166	0.4776	0.3739
1.1741	0.8023	0.9557	1.2213	0.6546	0.8375	1.5267	1.0628	0.7931	0.7808	0.8159
0.7767	0.8844	1.0406	0.8458	0.8923	0.6072	0.7395	1.1763	1.0069	0.7313	0.7825
0.7047	0.6858	0.5127	0.6987	0.7893	0.4098	0.6291	0.7881	0.6981	0.5472	0.5166
0.5661	0.9472	0.9714	0.6879	1.2134	0.5115	0.7434	0.8438	0.6864	0.6993	0.7016
0.8941	0.9079	0.9429	0.8774	0.7096	0.7409	0.5981	0.8857	0.594	0.7046	0.6772
0.9236	1.7284	1.6992	1.372	0.4156	1.4345	1.2098	0.998	1.0773	0.9469	1.0633
1.2349	0.8077	0.939	0.5447	0.3739	0.4723	0.3405	0.3647	0.3055	0.8207	0.5632

T. gondii-unr	G. lamblia-ur	L. donovanii-	N. gruberi-ur	T. vaginalis-u	A. niger-unre	B. dendrobat	C. albicans-re	C. albicans-u	S. cerevisiae-	S. cerevisiae-
0.9915	0.8905	1.1426	0.4878	0.701	0.7234	0.8388	0.4765	0.632	0.7068	0.6795
0.9911	1.0017	0.9704	0.9428	1.4363	0.9835	1.1068	0.7647	0.7815	1.0418	1.1075
0.9963	0.9235	0.7723	0.6806	0.7937	0.8294	0.8128	0.6208	0.6635	0.9025	0.8679

0.9903	1.2067	1.583	1.3668	1.9214	1.753	1.2005	1.451	1.6629	1.3587	1.3523
1.4013	1.0596	1.1163	1.2139	1.1074	1.2868	1.1388	1.1479	1.2784	1.0076	1.1164
0.9129	0.9855	0.6902	0.565	0.836	0.7579	0.8528	0.8069	0.8868	0.947	0.965
0.978	1.0776	0.8505	0.6753	1.2827	0.987	1.0017	0.8197	0.9344	0.8834	0.9127
0.6602	0.81	0.7112	0.8496	1.2664	0.6806	0.8282	0.79	0.8758	0.9393	0.9683
1.337	0.8556	1.0421	1.3602	1.0176	0.9126	1.06	0.5309	0.7776	0.9211	0.9553
0.7972	0.9317	0.8016	2.0436	0.8974	0.9145	1.0808	1.1499	0.9551	1.0817	1.0622
1.0564	1.0348	1.0496	1.0426	0.7632	0.9925	0.8194	0.8344	0.8442	0.87	0.9027
0.8904	0.614	0.4895	0.5036	0.3051	0.5111	0.5257	0.4177	0.598	0.7884	0.6154
0.8329	0.7299	0.7697	0.4906	0.5534	0.7525	0.7519	0.9879	0.8017	0.8771	0.8284
1.0733	0.898	1.1016	1.356	1.0205	0.8585	1.0005	1.2365	1.0606	1.1549	1.1037
1.2299	1.0696	1.0828	0.7048	0.7962	1.0988	1.3374	1.9312	1.3145	1.1004	1.0406
1.0028	0.8735	0.8947	0.6904	0.4879	0.7737	0.9171	0.8852	0.8863	0.8447	0.8242
0.9179	0.9954	0.804	0.6887	0.7363	0.9224	1.147	1.0837	0.8594	0.9017	0.8651
1.117	1.1365	1.6609	1.7549	1.3097	1.0123	1.0362	1.3385	1.4702	1.364	1.4407
1.0872	1.4279	0.9643	1.1261	0.853	1.3615	0.7662	0.8265	0.9124	0.8088	0.8023
0.8465	1.2774	0.6987	0.5777	0.4882	0.7098	1.0334	0.9482	0.6625	0.613	0.6343

T. gondii-unr	G. lamblia-ur	L. donovanii-	N. gruberi-ur	T. vaginalis-u	A. niger-unre	B. dendrobat	C. albicans-re	C. albicans-u	S. cerevisiae-	S. cerevisiae-
1.2109	0.9277	1.3346	0.6266	0.6922	0.7973	0.9143	0.4765	0.3829	0.8578	0.7112
0.8644	0.9799	0.8008	0.7135	0.6831	0.8742	1.1015	0.9361	0.8232	0.8444	0.8658
0.7585	0.8957	0.7732	0.5655	0.8373	0.5882	0.6715	0.4755	0.4898	0.6657	0.6749
1.124	1.0716	1.2315	1.2337	1.1661	1.4258	1.2584	1.213	1.3971	1.2217	1.2819
1.2078	0.9743	1.1495	1.2402	1.6984	1.3473	1.3347	1.2981	1.4371	1.118	1.1716
1.0036	0.9565	0.8803	0.7334	0.9345	0.752	0.9848	0.7684	0.8492	0.8976	0.9211
0.7528	1.0204	0.8271	0.7276	1.2091	0.7614	0.7414	0.6334	0.7823	0.8053	0.8459
0.6072	0.8365	0.7565	0.6181	0.9524	0.7182	0.7456	0.8013	0.8357	0.9813	0.9643
1.1576	0.8868	1.1213	1.3	0.835	1.0905	1.0273	1.0392	0.9709	1.0254	1.0503

0.9499	0.9896	0.7781	1.7571	1.2685	1.1028	0.9078	1.1814	1.054	1.4485	1.1629
1.1719	1.0015	0.9401	1.7831	0.5808	1.028	0.8332	0.7618	0.8799	0.813	0.8716
0.9089	0.6308	0.6694	0.4834	0.5147	0.5415	0.4954	0.3759	0.393	0.6248	0.58
1.16	0.7754	1.2564	0.6535	0.7698	0.8162	0.9048	0.8197	0.8546	0.8741	0.8557
1.0471	1.0136	1.413	1.0059	0.872	1.1281	0.9615	1.1241	0.9925	1.0592	1.0573
1.3241	1.2537	1.2801	1.0743	1.0885	1.1169	1.0794	1.264	1.1101	1.1434	1.084
1.1375	0.9187	1.2075	0.8314	1.11	1.0703	1.2563	1.058	1.0005	0.9043	0.959
1.0667	1.0558	0.9104	1.1127	0.8849	0.9652	1.0143	1.1498	0.9796	0.9904	0.9751
0.9916	1.1994	1.2087	1.4158	1.2429	1.0654	1.0441	1.5156	1.3998	1.2382	1.2382
1.2577	1.2608	1.1594	1.0101	0.7874	1.4778	1.0031	1.1072	1.3758	1.0824	1.1365
1.0407	1.5065	0.8887	0.5777	1.8054	0.7099	0.7046	0.5106	0.4138	0.8206	0.6898

T. gondii-unr	G. lamblia-ur	L. donovanii-	N. gruberi-ur	T. vaginalis-u	A. niger-unre	B. dendrobat	C. albicans-re	C. albicans-u	S. cerevisiae-	S. cerevisiae-
1.1911	0.931	1.0658	0.6791	1.1326	0.8034	0.6207	0.4765	0.5573	0.8736	0.6295
0.9393	0.9155	0.9164	0.905	0.9111	0.906	1.0057	0.7383	0.758	1.0056	0.9356
0.9376	0.9685	0.8193	0.6581	0.7888	0.7073	0.7893	0.5415	0.5572	0.7309	0.7752
1.095	1.0297	1.343	1.0776	1.0761	1.2782	1.0031	1.0978	1.2217	1.068	1.0898
1.2648	0.9808	1.2655	1.3062	1.1729	1.3965	1.3673	1.4912	1.4572	1.1963	1.3142
1.0443	1.0525	0.9707	0.8855	0.8205	0.7793	1.1634	0.8709	0.8929	1.035	1.0265
0.7175	0.9971	0.8677	0.831	0.867	0.8398	1.1066	0.7825	0.7577	0.7852	0.7944
0.7019	0.8928	0.7051	0.8138	1.3387	0.7467	0.803	0.6207	0.8528	0.8139	0.9506
1.1526	0.8676	1.0964	1.2369	0.8794	1.1556	1.0273	1.1521	0.999	1.0127	1.0512
0.9425	0.8989	1.0185	1.5859	0.9117	1.1771	1.0429	1.2601	1.0419	1.2519	1.1688
1.0575	1.0428	1.1233	1.5097	0.8132	0.957	0.9897	0.653	0.8006	1.141	1.1019
0.9966	0.7352	0.553	0.5266	0.4037	0.589	0.5156	0.543	0.4396	0.476	0.4614
0.9193	0.8982	0.941	0.6933	0.6176	0.8176	0.8709	0.9248	0.936	0.7897	0.7753
1.1794	0.9244	1.292	1.0345	0.9758	1.2119	0.942	1.2263	1.186	1.2757	1.2309
1.0889	1.2186	1.0144	0.8868	1.4744	0.9125	0.9063	0.9831	1.0007	1.0289	0.966

1.1762	0.9569	1.0373	0.9288	1.2701	1.0019	1.1281	1.1443	1.1911	1.0332	0.9851
0.9344	1.054	0.8723	0.9975	0.931	0.9911	0.9904	1.2574	0.9943	0.9236	0.911
1.0912	1.075	1.0323	1.0433	0.8913	1.1831	0.9549	1.0826	1.1955	1.1614	1.1931
1.0042	1.3351	1.1221	1.2367	0.9887	1.2069	1.0384	0.8889	1.1294	1.0449	1.0569
0.9279	1.604	0.6819	0.5942	0.8235	0.7071	0.9747	0.7294	0.5629	0.9096	0.7939

T. gondii-unr	G. lamblia-ur	L. donovanii-	N. gruberi-ur	T. vaginalis-u	A. niger-unre	B. dendrobat	C. albicans-re	C. albicans-u	S. cerevisiae-	S. cerevisiae-
1.4303	0.8952	1.1938	0.6454	0.7982	0.9419	0.8137	0.7489	0.4779	1.0563	0.7583
0.8897	0.9242	0.8859	0.9775	1.7632	0.9328	1.2576	0.6592	0.814	0.9675	1.0171
0.9265	0.9493	0.7908	0.6989	1.049	0.7498	0.7398	0.5019	0.5407	0.829	0.8318
1.1577	1.0486	1.4175	1.4483	1.4117	1.3044	1.082	0.9673	1.2681	1.1782	1.2012
1.0502	0.9184	1.0478	1.304	1.1055	1.1833	1.3097	1.1801	1.3579	1.1713	1.2197
1.1145	1.0557	0.9631	0.8552	0.7819	0.9	1.0375	1.0758	1.0132	1.0258	1.009
0.7306	0.8933	0.9527	0.7899	0.7992	0.9055	0.7611	0.4968	0.8011	0.7398	0.8256
0.6902	0.7809	0.7798	0.7898	0.8095	0.7884	0.8443	0.9931	0.8742	0.8525	0.8679
0.9295	0.7697	1.0219	1.0564	0.9487	0.9033	0.9183	1.0053	0.9	0.9283	0.9116
0.9613	1.0177	0.8457	1.2591	0.8709	1.0191	1.1105	0.8978	0.9836	1.0345	1.0845
1.0227	1.0317	1.121	1.3572	0.7859	1.0398	1.0725	1.2697	0.9147	0.8367	0.8815
0.7169	0.7638	0.5927	0.4949	0.5587	0.4503	0.46	0.9189	0.3345	0.5603	0.56
1.156	0.8346	0.8169	0.6228	0.7634	0.7256	0.7944	0.7777	0.8911	0.7866	0.79
0.9733	0.9186	1.3742	0.9573	0.8867	1.0214	0.955	1.2467	1.0737	1.0341	1.0244
1.1548	1.3908	0.9662	0.8663	1.445	0.8934	0.9975	1.0358	0.9635	1.0766	1.0281
0.9741	0.9007	1.1008	0.9047	0.8227	1.0486	1.1853	1.4466	1.1609	1.0596	1.0461
1.0208	1.1861	0.9144	1.0157	0.9078	1.1052	0.9904	1.2326	1.0353	1.0856	0.9606
1.0969	1.1539	1.1235	1.1546	0.8783	1.0565	0.8998	1.1416	1.2959	1.2132	1.3351
1.1317	1.2635	1.064	1.2702	1.2769	1.4352	1.109	1.1072	1.2383	1.0543	1.0943
1.0395	1.5582	0.9055	0.6272	0.4937	0.6607	0.775	0.3647	0.595	0.9788	0.8118

S. pombe-rev	S. pombe-un	A. aegypti-ur	D. rerio-revie	D. rerio-unre	D. melanogas	D. melanogas	G. gallus-revi	G. gallus-unr	H. sapiens-re	M. mulatta-u
0.2729	0.6008	0.6542	0.362	0.4543	0.6663	0.7908	0.49	0.5208	0.3461	0.4592
1.6682	1.8094	1.8249	3.3175	3.1588	1.8763	1.6008	3.5969	2.9109	3.159	3.2613
0.9959	0.6159	0.7765	1.0149	0.998	0.8966	0.7709	1.2544	1.3251	1.27	1.1588
2.4561	2.8991	1.6161	1.7221	1.6308	2.1085	1.8751	1.5453	1.4415	1.427	1.3389
1.0703	1.1513	0.9503	1.0502	0.8765	0.9359	0.9299	0.7118	0.9213	0.7321	0.8294
0.7116	0.699	0.7391	0.5443	0.5569	0.7315	0.6719	0.6868	0.5786	0.5832	0.6908
1.3041	0.8538	1.3039	1.3343	1.3069	1.1172	1.1742	1.0874	0.9956	1.4777	1.1642
0.9685	0.6152	1.0638	1.2368	1.1606	0.9004	0.9928	1.3035	1.3883	1.5792	1.2561
1.0898	0.7181	0.9982	1.2529	0.9206	1.068	0.9752	0.8823	0.7568	0.9761	0.9127
0.7542	0.7712	0.6348	0.4139	0.488	0.5201	0.7147	0.436	0.5867	0.4938	0.5012
0.4391	0.45	0.659	0.5205	0.4027	0.5614	0.7668	0.5654	0.6076	0.4105	0.4439
0.4972	0.7165	0.7814	0.8381	1.167	0.8306	0.7157	0.6338	0.6742	0.7176	0.8281
0.3304	0.5421	0.5223	0.3006	0.4543	0.5435	0.6065	0.3331	0.3737	0.3245	0.3634
0.9152	0.4578	1.1865	0.6034	0.8314	0.9997	1.1749	0.6545	0.7398	0.742	0.7762
0.765	0.6987	1.1165	0.6584	0.7227	1.1239	1.0395	0.4731	0.5196	0.5086	0.5375
0.4233	0.539	0.7281	0.4226	0.4459	0.4771	0.5506	0.2424	0.3212	0.3322	0.3922
0.5855	0.7431	0.7565	0.4677	0.5457	0.7283	0.7603	0.451	0.6099	0.4787	0.5653
0.716	0.5904	0.8824	0.5062	0.7405	0.6048	0.8718	0.8184	0.9354	0.7317	0.775
0.9257	1.1955	0.7289	0.8683	0.8393	0.9487	0.9202	0.8444	0.8751	0.7674	0.8575
0.3433	0.3834	1.0582	0.5568	0.8529	0.9314	1.1299	0.445	0.8692	0.9653	1.0381

S. pombe-rev	S. pombe-un	A. aegypti-ur	D. rerio-revie	D. rerio-unre	D. melanogas	D. melanogas	G. gallus-revi	G. gallus-unr	H. sapiens-re	M. mulatta-u
0.7148	1.0014	1.155	0.8184	0.9285	0.8713	0.9579	0.9611	1.0314	0.7605	0.8789
0.8741	0.8519	0.8742	1.4203	1.3195	1.1511	1.001	1.6659	1.7104	1.5865	1.5217
0.7789	0.6755	0.6902	1.0432	0.9069	0.704	0.6931	1.2864	1.0883	1.0874	1.1047

1.4195	1.6984	1.3831	1.4377	1.3334	1.2276	1.2472	1.5347	1.2812	1.3334	1.2903
1.126	1.1952	1.1066	1.2653	1.0827	1.3629	1.1481	1.0493	0.9399	1.087	1.1317
0.9213	0.752	0.7438	0.898	0.892	0.6896	0.764	0.7063	0.8042	0.8444	0.9115
0.8218	1.4111	0.9856	1.2505	1.207	1.1809	0.9951	0.8795	0.8839	1.007	0.939
1.1231	0.8169	0.7814	0.9982	1.0375	0.8796	0.846	1.0309	0.9785	0.8733	0.9265
1.1156	1.3073	1.0406	0.9597	1.0081	0.9709	1.1107	0.9228	1.0408	0.8916	0.8179
1.0339	1.1651	0.8435	0.785	0.8224	0.708	0.8595	0.6919	0.8185	0.8544	0.8012
0.6248	0.9845	0.8677	0.6856	0.7307	0.8983	0.9429	0.8304	0.7855	0.7377	0.7072
0.5433	0.5608	0.7687	0.8381	0.8775	0.9061	0.869	0.8275	0.7148	0.7508	0.8261
0.8793	1.0843	0.598	0.4689	0.5949	0.7375	0.7926	0.3997	0.5156	0.4321	0.45
1.1581	0.8902	1.1774	0.9515	0.8906	1.3184	1.4211	0.8772	0.7721	0.8508	0.8332
1.2223	1.1824	1.505	0.823	1.4295	1.2501	1.2383	0.6712	0.8731	0.6977	0.7625
0.9026	0.5784	0.9508	0.6113	0.6616	0.8452	0.9271	0.5683	0.4709	0.4865	0.5374
0.857	0.7649	1.0101	0.9004	0.9252	0.9245	0.9753	0.8037	0.8612	0.9684	0.9197
1.1933	1.0542	1.2725	1.0305	1.0752	1.3181	1.2309	1.3538	1.3551	1.2552	1.2019
0.8648	1.0422	1.0226	0.8937	0.7331	0.8014	0.776	0.9325	1.2275	1.2404	1.2696
0.4806	0.5751	1.0814	0.6154	1.0087	0.7097	1.0669	1.027	0.9447	1.0864	1.1799

S. pombe-rev	S. pombe-un	A. aegypti-ur	D. rerio-revie	D. rerio-unre	D. melanogas	D. melanogas	G. gallus-revi	G. gallus-unr	H. sapiens-re	M. mulatta-u
0.6498	0.5341	0.8597	0.9286	0.8875	0.7047	0.8293	0.6784	1.4278	0.7605	0.8367
0.9141	0.8821	0.8084	0.9382	1.0119	0.8425	0.8391	1.2797	1.2629	1.2551	1.2771
0.6316	0.9537	0.6439	0.8448	0.806	0.578	0.6396	1.0944	1.1286	0.9179	1.0394
1.1262	1.3587	1.1045	1.2295	1.2259	1.1018	1.048	1.1364	0.9606	1.1005	1.1608
1.1818	1.3925	1.0305	1.2084	1.0291	1.1576	1.1223	0.9246	0.7823	0.9224	0.9902
0.8832	0.8579	1.0517	1.1484	1.0514	0.9525	0.8987	0.9266	1.0707	0.8412	0.8766
0.761	0.8656	0.7017	0.8186	0.7997	0.9261	0.7526	0.5437	0.6604	0.6406	0.679
0.9306	0.6253	0.7263	0.8131	0.8191	0.7788	0.7977	0.8591	0.8506	0.8238	0.8784
1.2886	1.3257	1.0998	0.9064	0.9416	0.9806	1.1363	0.8214	0.8128	1.1115	0.8236

1.1088	1.0338	0.8236	0.8421	0.8959	0.8654	0.9172	0.7772	0.8184	0.9919	0.841
0.9035	0.9563	0.8606	0.876	0.7974	0.9242	0.8577	0.8657	1.0305	0.6938	0.7521
0.5985	0.4673	0.8143	0.9598	0.9432	0.7268	0.8246	0.8099	0.8492	0.7736	1.1264
1.0697	1.0585	0.8668	1.0581	0.8447	1.1956	0.9908	0.7861	0.7609	0.6171	0.6719
1.2618	0.8394	1.1135	0.9805	1.0139	1.1453	1.3018	0.7962	0.8015	0.8948	0.9006
1.0768	1.0749	1.4466	1.2802	1.1062	1.509	1.3455	1.1443	0.9653	1.0778	0.9131
0.9586	0.8019	1.5319	0.8981	1.1954	1.2042	1.2197	0.6519	0.6755	0.8371	0.7022
1.0082	1.0053	1.2219	0.9353	1.0601	1.1771	1.2034	1.1207	1.2278	1.148	1.0758
1.1072	1.0331	1.1221	1.2353	1.1699	1.0428	1.1084	1.6061	1.3018	1.439	1.3798
1.0028	1.3692	0.8457	1.0268	0.9737	1.0266	0.9331	1.3658	1.2383	1.2448	1.2706
0.6523	0.4313	1.1276	0.8498	1.2614	1.0645	1.2423	1.3008	1.2284	1.3103	1.2592

S. pombe-rev	S. pombe-un	A. aegypti-ur	D. rerio-revie	D. rerio-unre	D. melanogas	D. melanogas	G. gallus-revi	G. gallus-unr	H. sapiens-re	M. mulatta-u
0.7278	1.2029	1.1174	1.1017	0.8606	0.7816	0.9854	0.9611	0.902	0.8287	0.9381
0.8095	0.9882	0.903	1.0834	1.0664	0.9814	0.8724	1.3085	1.33	1.2401	1.275
0.8486	0.8152	0.8249	1.1679	1.0716	0.8448	0.7598	1.408	1.4969	1.2933	1.3086
1.0855	1.4467	1.0782	1.0933	1.1212	0.9759	1.0388	1.1045	1.1122	1.0443	1.0743
1.1539	1.3167	0.9763	1.0249	1.0209	1.2069	1.114	0.976	0.8147	1.0857	1.0671
0.8864	0.9432	1.1029	1.0668	1.149	0.9563	0.9762	1.121	0.954	1.0425	1.0053
0.8539	0.7712	0.7979	0.9669	0.7848	0.8071	0.7929	0.7356	0.7562	0.6972	0.8098
1.0414	0.9083	0.8394	0.9349	0.9381	0.9283	0.871	0.8413	0.8198	0.8113	0.8379
1.2702	1.124	0.9853	0.9774	0.909	1.034	1.0441	0.8113	0.6268	0.9222	0.8885
1.2537	0.8702	0.8222	0.8064	0.8244	0.8042	0.9808	0.8245	1.017	0.9911	0.8228
0.9203	1.4071	0.697	0.711	0.8464	0.6823	0.8566	0.53	0.744	0.6953	0.6685
0.4788	0.4052	0.7839	1.0139	0.9333	1.0477	0.8397	0.8099	0.9	0.7841	0.9245
0.8009	0.8266	1.0636	0.6974	0.7024	0.8928	0.8292	0.6262	0.5367	0.6793	0.6139
1.3862	1.0308	1.0932	1.2532	1.406	1.5152	1.3157	1.1268	0.8377	1.0339	1.031
0.9936	0.735	1.1923	1.1156	0.9803	0.9345	1.0574	0.8912	0.9745	0.8262	0.8329

1.0022	0.9208	1.2719	0.8226	0.9089	1.1406	1.1342	0.6101	0.9523	0.7252	0.6806
0.8919	0.9039	1.1411	0.9249	1.0109	1.0766	1.1462	0.951	1.0229	0.9938	1.0151
1.1972	1.0549	1.175	1.0425	1.1687	1.1972	1.2063	1.4838	1.3665	1.2763	1.3005
0.8567	1.1145	0.9023	0.8303	0.6987	0.8014	0.8297	0.9032	0.9813	1.1056	1.0347
0.7724	0.6714	0.9194	1.348	1.1625	0.998	1.4462	1.3693	1.162	1.4042	1.2811

S. pombe-rev	S. pombe-un	A. aegypti-ur	D. rerio-revie	D. rerio-unre	D. melanogas	D. melanogas	G. gallus-revi	G. gallus-unr	H. sapiens-re	M. mulatta-u
0.9877	0.7343	1.0392	0.9286	0.9704	1.0507	1.1379	1.451	1.1432	1.0129	0.9531
0.8464	1.0103	1.0012	1.1248	1.0449	1.1048	0.9829	1.4468	1.4839	1.2116	1.2636
0.7828	0.8444	0.7312	1.0205	0.9363	0.867	0.8149	1.2672	1.2093	1.1179	1.1812
1.1975	1.3982	1.0789	1.1774	1.1245	1.1969	1.027	1.2373	1.0517	1.1422	1.0974
1.1191	1.3487	1.0461	0.9806	0.9371	0.977	0.9223	0.6384	0.6971	0.9799	0.9005
0.9086	0.8791	1.4109	1.143	1.1698	1.0516	1.1494	0.959	0.9878	1.017	1.0208
0.7789	0.83	0.8277	1.0056	0.8728	0.8581	0.8473	0.8955	1.0345	0.8696	0.8467
0.9364	0.595	0.8529	0.9203	1.116	0.8066	0.8549	0.8413	0.8425	0.9475	0.841
0.9978	1.4177	0.8991	0.8708	0.8069	1.1699	1.0444	0.6186	0.7526	0.679	0.6794
0.984	1.1815	0.8577	0.8207	0.8934	0.8479	0.8514	0.7866	0.9812	0.8808	0.8804
0.819	1.097	0.6828	0.6348	0.7219	0.6564	0.7719	0.7774	0.7463	0.6423	0.6835
0.4972	0.3427	0.7283	0.757	0.8944	0.8212	0.7179	0.6338	0.6439	0.7404	0.7936
0.8793	0.7745	0.8649	0.7455	0.878	0.8462	0.845	0.5463	0.5846	0.7346	0.62
1.4602	1.0556	1.0476	1.0792	1.0262	1.2122	1.2245	0.9717	1.0771	0.9844	0.9423
1.031	0.8241	1.0376	1.0973	1.1024	1.1491	1.1122	0.9793	0.9139	0.8367	0.8741
1.0707	0.7888	1.1611	0.9585	0.9481	1.0588	1.2085	0.5767	0.6515	0.7228	0.6839
1.016	0.8669	1.2247	1.0191	1.05	1.1452	1.1848	1.1877	1.0102	1.069	1.155
1.162	1.2229	1.0974	1.1992	1.1752	1.0261	1.1327	1.3461	1.395	1.3003	1.3008
0.8485	1.2568	0.8244	0.8747	0.8583	0.7104	0.819	1.0721	1.0067	1.1487	1.1955
0.8582	0.4313	0.9426	0.8498	0.9245	0.9536	1.1786	0.8216	1.0865	1.2016	1.2423

M. gallopavo	M. musculus·	M. musculus·	T. castaneum	X. tropicalis-r	X. tropicalis-u	E. hellem-uni	E. bieneusi-u	N. parisii-unr	N. bombycis-	V. corneae-ui
0.6235	0.3337	0.4934	0.7714	0.3617	0.3631	0.3349	0.7078	0.8184	0.5478	0.4677
2.5885	3.5434	2.7386	2.2922	3.8678	3.5629	1.2066	1.1762	1.2691	1.3947	1.3715
1.1196	1.1666	1.1067	1.0828	1.1287	1.1819	1.1725	1.1704	0.9682	0.7559	0.8556
1.6415	1.4232	1.3215	1.9291	1.6758	1.591	1.064	1.1968	0.9276	1.3076	1.2089
1.4033	0.7882	0.9185	1.0285	0.8469	0.7553	0.7251	0.9528	0.708	1.0417	0.8529
0.6556	0.5577	0.7084	0.7141	0.5574	0.6295	0.5592	1.0031	0.6566	0.7534	0.789
1.1626	1.2108	1.398	1.1758	1.244	1.2552	1.6094	1.1867	1.4286	1.0593	1.2193
1.2496	1.411	1.4059	0.98	1.4011	1.3451	1.6466	1.0721	1.2107	0.7906	1.2201
0.8655	0.8378	1.0046	1.0135	0.782	0.8104	1.5613	0.9804	1.3529	1.2588	1.192
0.5322	0.423	0.5147	0.6225	0.4828	0.4839	0.8496	1.2698	1.6401	1.1246	1.3503
0.4514	0.3956	0.4163	0.7108	0.4491	0.5722	0.7247	0.9856	1.9475	1.1648	1.2449
0.6564	0.7327	0.8655	0.6415	0.5726	0.5831	0.2984	0.4854	0.4158	0.6735	0.3084
0.2531	0.2775	0.4382	0.5803	0.3696	0.3771	0.3154	0.9091	0.6052	0.6064	0.436
0.8647	0.8003	0.8569	0.9151	0.5831	0.7507	1.5841	1.1219	1.6785	1.3611	1.2475
0.6085	0.4912	0.7141	0.8873	0.4954	0.6065	0.6258	1.1579	0.822	0.932	0.8013
0.382	0.3046	0.4578	0.4692	0.3655	0.3308	0.7681	0.9072	0.4046	0.9563	0.7166
0.5894	0.4974	0.588	0.5987	0.4059	0.5437	0.6035	0.7217	0.6028	0.7356	0.8119
0.7433	0.6983	0.7905	0.8322	0.5471	0.6058	1.0049	0.8388	1.3825	1.1105	0.9939
0.8985	0.8664	0.8565	0.92	0.8389	1.0142	1.0153	0.9025	0.8593	0.9564	1.6222
0.788	1.0079	1.0175	1.0196	0.985	0.811	0.509	0.9333	1.0281	0.8864	0.8296

M. gallopavo	M. musculus·	M. musculus·	T. castaneum	X. tropicalis-r	X. tropicalis-u	E. hellem-uni	E. bieneusi-u	N. parisii-unr	N. bombycis-	V. corneae-ui
1.0949	0.8344	0.8176	0.9629	0.8068	0.8378	0.8758	0.9101	1.1199	0.8739	0.7249
1.3805	1.6793	1.3945	0.9956	1.6357	1.5474	0.7894	0.6992	0.7481	0.799	0.7434
0.9109	1.1068	1.07	0.8611	1.3054	1.1763	0.9715	0.9828	0.8169	0.4462	0.8775

1.44	1.3662	1.3614	1.3072	1.2917	1.3813	1.0574	0.9705	0.9575	1.0652	1.2032
1.0206	1.1544	1.1936	1.1038	1.2761	1.0719	1.1704	0.9469	1.0419	0.9866	1.2694
0.8279	0.7705	0.8516	0.9268	0.8019	0.8344	0.7736	0.9906	0.9529	0.8687	0.7808
1.2948	0.9436	0.8949	1.0641	1.0983	1.0836	0.9919	0.9681	0.7901	0.7766	0.8388
0.9185	0.9421	0.9229	0.9958	1.1539	1.0774	1.3074	1.2288	1.1455	0.8228	1.1803
1.5872	0.7557	1.031	0.9392	0.8255	0.8403	1.0689	1.2458	1.2195	1.1438	1.1759
0.784	0.7786	0.8459	0.9665	0.6932	0.7868	1.0562	0.8791	1.2687	1.1656	1.366
0.8654	0.7148	0.7548	0.996	0.4491	0.84	0.7537	0.7755	0.8666	1.002	1.1951
0.7182	0.7273	0.9186	0.4937	0.3817	0.7424	0.4564	0.638	0.6274	0.6936	0.6373
0.5511	0.4466	0.5311	0.7223	0.642	0.5711	0.7541	0.767	0.9604	0.8351	0.7872
0.912	0.8087	0.8926	1.0916	0.8052	0.8932	1.2765	1.3166	1.0499	1.0268	1.1393
0.9346	0.8128	0.8357	1.0422	0.9133	0.8983	1.0183	1.2544	0.9301	1.5265	1.0037
0.5332	0.4732	0.6696	0.7936	0.5726	0.5728	0.9091	0.9231	1.2462	1.1637	1.0254
0.8897	0.9089	0.9111	1.0049	0.9634	0.9278	0.9363	0.8801	0.7577	1.1534	0.8357
1.0746	1.3001	1.186	1.2917	1.0395	1.1746	1.2196	0.96	1.5762	1.04	1.0155
0.785	1.1427	1.033	0.92	1.0568	0.9208	0.6158	0.7154	0.6234	0.8931	0.8187
1.0425	1.2201	1.3475	0.8178	0.8295	0.9406	1.8904	1.1555	0.9995	0.9249	1.0785

M. gallopavo	M. musculus	M. musculus	T. castaneum	X. tropicalis-r	X. tropicalis-u	E. hellem-un	E. bieneusi-u	N. parisii-unr	N. bombycis-	V. corneae-u
0.9564	0.8662	0.9026	0.86	1.1406	0.9352	0.6955	0.6741	0.7861	0.9652	0.6781
0.9679	1.3949	1.1812	0.8695	1.2471	1.1804	0.9473	0.7156	0.709	0.9532	1.1936
0.882	0.9746	0.9642	0.7141	0.9619	0.9013	0.7956	0.9678	0.7996	0.6876	0.8336
1.0953	1.1985	1.1389	1.1969	1.1242	1.1282	0.9008	1.2521	1.1508	0.9814	1.0264
0.8501	0.9724	0.9566	1.0682	1.2083	0.9237	0.9032	0.9883	1.1388	1.138	1.2694
1.0638	0.8845	0.9549	0.9897	0.9975	0.9655	0.8732	1.1097	1.0135	0.7658	0.9679
0.6894	0.7053	0.7	0.7746	1.0087	0.709	0.9451	0.8494	0.7622	0.9048	0.8215
0.7994	0.8215	0.8623	0.8196	0.9643	0.9074	1.3074	0.7931	1.0327	0.896	1.0609
0.8549	0.8099	0.858	1.081	0.9558	0.9324	1.0689	1.1259	1.0258	1.1127	0.9503

0.8969	0.8597	1.0196	0.9169	0.7923	1.0197	1.4006	0.8888	1.3059	1.1985	1.4288
0.8466	0.7426	0.8713	0.905	0.4266	0.799	0.8697	0.8402	0.8852	0.9644	1.0209
0.9302	0.8172	0.9944	0.6248	0.4056	0.7669	0.6143	0.6241	0.569	0.7841	0.7606
0.8639	0.6873	0.8738	0.8696	0.7782	0.9171	0.6307	0.7599	0.7148	0.9096	0.7024
0.9938	0.8715	0.8993	1.1267	0.7959	0.9711	1.2896	1.3201	1.1941	1.0006	1.1621
0.9739	0.9296	1.062	1.3124	1.0681	0.9371	0.944	1.4112	1.0052	1.3731	0.8772
0.9064	0.6676	0.8313	1.0871	0.7919	0.8758	1.1134	0.885	0.9229	0.9815	0.8856
1.4437	1.1006	1.0836	1.1537	1.0906	1.0757	1.1394	0.9153	1.055	1.2283	1.0841
1.2808	1.3211	1.2045	1.1911	1.2036	1.2747	1.0221	1.0345	1.4366	0.9048	0.9939
1.0041	1.3263	1.1339	0.906	1.2529	1.2447	0.8322	1.1556	0.8874	0.8439	1.0158
1.4239	1.36	1.4726	0.855	0.9331	1.3533	0.5817	0.8	1.0566	0.6552	0.6637

M. gallopavo	M. musculus	M. musculus	T. castaneum	X. tropicalis-	X. tropicalis-	E. hellem-un	E. bieneusi-u	N. parisii-unr	N. bombycis-	V. corneae-u
0.9426	0.9377	0.8985	0.9286	0.9459	0.9327	0.6182	0.9944	0.8184	0.913	0.8886
0.977	1.3215	1.1407	0.9122	1.2109	1.1246	0.733	0.8474	0.8656	0.7219	0.9528
1.0984	1.3351	1.1404	0.8589	1.3643	1.3546	0.5946	0.7881	0.7478	0.7034	0.7349
1.1315	1.0611	1.0097	1.0984	1.2289	1.1016	0.9726	0.9507	0.8217	0.8887	1.0492
0.929	0.956	1.086	1.0427	0.8695	0.9761	1.094	1.0302	1.0687	1.1197	1.1405
0.9107	1.0196	0.9892	1.097	1.1344	1.1168	0.9805	0.9344	1.1044	1.0046	0.8622
1.0491	0.7462	0.7692	0.8305	0.7733	0.9029	1.0761	0.8747	0.6385	0.7464	0.8302
0.8537	0.8324	0.84	0.9482	1.1539	0.9496	1.1224	0.8667	1.1831	0.814	1.2333
0.754	0.8049	1.3726	1.0204	0.6082	0.8934	0.8287	1.2419	0.8574	0.9977	0.9181
1.4101	0.861	0.8832	0.9679	1.0275	0.8386	1.1481	1.2018	1.5534	0.9194	1.4288
0.8655	0.694	0.7096	0.8509	0.6512	0.7363	1.1885	0.8241	0.9598	1.0646	1.1951
0.9714	0.9262	0.9439	0.7029	0.8112	0.8649	0.5968	0.6381	0.7733	0.9449	0.6167
0.6306	0.6179	0.6254	0.7726	0.7976	0.6938	0.809	0.6821	0.8815	0.9043	0.763
1.0833	0.9876	0.9884	1.1092	0.9162	1.0472	1.3158	1.2214	1.2955	1.1645	1.1848
1.2725	0.872	0.9007	1.0998	0.8205	0.9558	1.0713	1.1402	0.8643	1.2044	1.1724

0.6547	0.6989	0.8505	1.0691	0.865	0.8554	1.1487	1.0725	0.9108	1.151	0.9788
1.0447	1.0483	1.054	1.0758	0.9876	1.0125	1.0548	1.1375	0.9683	1.1489	0.9647
1.1299	1.3347	1.2154	1.1991	1.1489	1.1898	1.1423	0.839	1.6573	1.1575	1.0479
0.971	0.9926	0.9865	0.9002	0.7626	0.8003	0.8488	0.87	0.8481	0.9424	0.6822
1.1571	1.4854	1.6223	0.9718	1.4516	1.3676	1.236	0.8007	1.2565	1.002	0.9955

M. gallopavo	M. musculus	M. musculus	T. castaneum	X. tropicalis-r	X. tropicalis-u	E. hellem-unr	E. bieneusi-u	N. parisii-unr	N. bombycis-	V. corneae-ur
1.3378	1.0383	1.0011	0.9514	1.3075	0.9968	0.7985	0.7921	1.1199	0.9652	1.0289
1.12	1.3527	1.1032	0.9518	1.22	1.1411	0.9473	0.9788	0.8343	0.6588	0.9004
0.9207	1.1521	1.0669	0.8311	1.3545	1.1077	0.7119	0.8328	0.9812	0.7821	0.691
0.9638	1.1365	1.0798	1.1079	1.0962	1.0399	0.9334	0.9152	0.8677	0.9485	1.0321
0.8398	0.9384	0.9761	0.9083	0.926	0.8561	1.2085	0.9587	1.1789	0.8994	1.3487
1.0217	0.9919	1.0233	1.2237	1.0855	1.1472	0.9498	1.0282	0.8418	0.7823	0.8459
1.511	0.863	0.926	1.0015	1.0311	0.8895	0.7673	1.1305	0.7462	0.7954	0.7783
0.9081	0.8662	0.9204	0.9121	1.0879	0.948	1.1594	0.9546	1.118	0.858	1.0012
1.0392	0.6325	0.8255	0.9129	0.8834	0.8104	1.0328	0.9804	0.8352	1.1625	0.9826
1.0716	0.8148	0.9187	0.9359	0.7551	1.1053	1.4466	1.0256	1.2502	1.2723	1.1933
0.7465	0.6477	0.7763	0.7649	0.6736	0.6867	1.0146	1.0502	0.587	0.8141	0.8466
0.5884	0.7355	0.8302	0.6248	0.5487	0.6762	0.4037	0.7906	0.4888	0.6534	0.4111
0.6754	0.6288	0.775	0.8175	0.5447	0.7175	0.9461	0.8594	1.1139	0.7819	0.9204
1.0389	0.9415	0.9678	1.018	1.0273	1.0994	1.3288	1.0405	1.3203	1.0378	1.2703
0.9623	0.8832	0.916	1.1698	0.9598	0.9644	1.0077	1.375	0.9864	1.3923	1.0121
0.7555	0.7057	0.9164	1.0671	0.7188	0.8533	0.9866	1.0563	1.0876	1.2978	0.9846
1.068	1.1465	1.1204	1.1353	0.9573	1.0687	1.224	1.0597	0.9208	1.1988	1.1557
1.1882	1.2844	1.2555	1.1374	1.1489	1.2649	0.919	0.6897	1.4861	0.9871	0.9507
0.8962	1.1131	0.9969	0.9445	0.9697	0.9996	0.7323	1.0896	0.7357	0.8861	0.8945
1.1572	1.2057	1.0826	0.8922	1.1405	0.9885	0.8725	0.8	0.9424	0.4625	1.3274

A. thaliana-re	A. thaliana-u	C. variabilis-u	O. sativa-revi	O. sativa-unr	O. tauri-unre	P. patens-unr	P. trichocarpa	Z. mays-unreview
0.2385	0.5112	0.5348	0.2517	0.383	0.4458	0.735	0.428	0.579
3.7558	2.8571	1.5991	3.6189	3.4103	1.5332	2.3441	3.2679	2.7716
1.2739	1.3301	0.8093	1.1447	1.2101	0.9174	1.3801	1.4452	1.143
1.2542	1.1369	1.5653	1.3178	0.9846	2.6196	0.9122	1.0154	1.0055
0.8929	0.9171	1.0586	0.6698	0.7972	1.3464	0.8044	0.8757	0.8222
0.7558	0.8582	0.8306	0.5755	0.8279	0.4649	0.9793	0.7949	0.7436
1.3617	1.178	0.8785	1.4632	1.1991	1.0917	1.3936	1.3048	1.2363
1.7029	1.5039	0.9219	1.83	1.5821	0.5781	1.8147	1.7476	1.3907
0.7781	0.792	0.8576	0.4002	0.4684	0.788	0.6886	0.6625	0.6199
0.5251	0.6723	1.1968	0.5601	0.6	0.3338	0.8017	0.6993	0.7532
0.2352	0.5655	0.7823	0.2499	0.4719	0.7012	0.5323	0.517	0.654
0.9488	0.7531	0.2583	0.9417	0.5093	0.2381	0.3451	0.7025	0.7326
0.3702	0.5117	0.3465	0.2379	0.286	0.3339	0.5956	0.437	0.5556
0.9152	0.8369	0.7856	0.4876	0.5437	0.5657	0.7179	0.8526	0.6256
0.3336	0.5544	0.5273	0.2304	0.2554	0.7546	0.5431	0.453	0.4759
0.4147	0.553	0.4705	0.3204	0.2722	0.5107	0.4319	0.5204	0.4691
0.2941	0.4823	0.705	0.2724	0.3572	0.3938	0.4459	0.4722	0.5353
0.6428	0.7445	0.9293	0.4844	0.65	1.2598	0.8737	0.5928	0.6775
0.4834	0.7132	1.2319	0.6137	0.9255	1.8631	0.8521	0.6726	0.9169
0.3279	0.5783	0.6081	0.3195	0.6595	0.3928	1.5688	0.5429	0.6644

A. thaliana-re	A. thaliana-u	C. variabilis-u	O. sativa-revi	O. sativa-unr	O. tauri-unre	P. patens-unr	P. trichocarpa	Z. mays-unreview
0.7429	0.9185	0.9407	0.7153	0.8332	0.7119	0.9505	1.0407	1.0382
0.9834	1.1663	1.2117	1.4682	1.4153	1.3532	1.0443	0.9962	1.319
0.8232	0.9449	0.9038	1.0205	1.044	0.6824	0.9316	0.9661	1.0262

1.7025	1.476	1.3908	1.901	1.4158	1.5389	1.5954	1.572	1.4777
1.586	1.3494	1.2184	1.2978	1.2983	1.7525	1.2654	1.4493	1.2762
0.6422	0.7676	0.8795	0.6143	0.7873	0.7455	0.8796	0.7587	0.7213
0.8014	0.8281	0.9646	0.7927	0.8001	1.0529	0.7837	0.8178	0.7903
0.9358	0.9289	0.8048	0.7698	0.9112	0.458	0.9838	0.9799	0.807
1.2544	1.0269	1.0607	0.7559	0.7469	0.8065	1.3103	0.9848	0.778
0.8323	0.8357	0.9076	0.8567	0.8281	0.65	0.8411	0.8112	0.8499
0.7217	0.7643	0.8927	0.6727	0.7849	0.9109	0.9331	0.7495	0.8799
1.2244	1.027	0.5222	1.1952	1.152	0.4903	0.8906	1.0396	1.108
0.5432	0.6069	0.4807	0.3489	0.5799	0.5032	0.6207	0.6247	0.6191
1.1342	1.104	1.082	0.9086	0.8665	0.7142	0.8751	1.0258	0.8527
0.8185	0.8538	0.6703	0.635	0.6825	0.6491	0.7511	0.9587	0.7441
0.9328	0.8176	0.7986	0.636	0.7284	0.7317	0.9293	0.8927	0.7359
0.7954	0.8033	0.8442	0.7158	0.7303	0.8302	0.8616	0.8343	0.7965
1.2517	1.2395	1.1624	1.4028	1.2672	1.6603	0.9983	1.2224	1.2246
0.7728	1.0097	1.1656	1.0928	1.1661	1.1881	1.0993	0.8258	1.1914
0.5371	0.7389	0.7407	0.7632	0.9404	0.5461	0.8545	0.7892	0.9106

A. thaliana-re	A. thaliana-u	C. variabilis-u	O. sativa-revi	O. sativa-unr	O. tauri-unre	P. patens-unr	P. trichocarpa	Z. mays-unreview
0.9267	0.9793	0.9923	0.9007	0.8926	0.8557	1.088	1.1101	1.1113
0.8639	1.1324	1.0169	1.5959	1.3842	1.4224	0.9355	0.9624	1.3387
0.7711	0.8709	0.7899	1.124	1.0791	0.7243	0.9359	0.8444	0.9939
1.5413	1.3092	1.1783	1.5015	1.2482	1.1563	1.1245	1.2485	1.2768
1.3135	1.1853	1.3626	1.0327	1.1702	1.4226	0.9881	1.2735	1.1624
0.822	0.8861	0.9283	0.6983	0.9063	0.9331	0.8863	0.8165	0.8431
0.6573	0.7398	0.7975	0.6325	0.7149	1.1176	0.7372	0.6747	0.7375
0.9939	0.8975	0.7727	0.8603	0.8708	0.6124	1.0507	0.9792	0.8351
1.0636	0.9513	0.9196	0.7622	0.6896	0.8697	1.1273	0.9892	0.7414

1.1229	0.9121	0.988	1.0017	0.9547	0.6809	1.8259	1.0816	0.9622
0.828	0.9642	0.9528	0.7688	0.9327	0.863	0.8614	0.9187	0.9425
0.8891	0.8768	0.5054	0.7968	0.7926	0.5371	0.7711	0.9282	0.8304
0.6455	0.8348	0.6763	0.6105	0.6364	0.7694	0.7436	0.7867	0.7052
1.0685	1.0527	1.0856	0.7934	0.8703	0.7048	1.0927	1.0678	0.8389
0.9698	1.008	0.985	0.8092	0.7558	0.7942	0.8555	1.0458	0.8507
1.0009	0.9224	0.9151	0.7915	0.6954	0.8638	0.8108	1.0092	0.7745
1.0107	0.9621	1.0825	0.8347	0.9057	0.7749	0.987	1.029	0.9401
1.1378	1.1272	1.1188	1.3176	1.1953	1.518	1.1585	1.0393	1.1125
0.9198	1.0709	1.3453	1.1222	1.2645	1.1909	0.8864	1.0535	1.2336
0.6163	0.8008	0.8732	0.5502	0.9872	0.5653	0.9338	0.8569	0.9802

A. thaliana-re	A. thaliana-u	C. variabilis-u	O. sativa-revi	O. sativa-unr	O. tauri-unre	P. patens-unr	P. trichocarpa	Z. mays-unreview
0.9189	0.9915	1.1212	0.8875	0.8727	0.6903	1.0772	1.1442	1.0418
0.854	1.1359	1.0147	1.4436	1.3839	1.3471	0.9652	0.9461	1.2748
0.8721	1.0193	0.9644	1.3281	1.2383	0.8129	1.1121	0.9003	1.1181
1.4104	1.2456	1.0971	1.2245	1.1782	1.1777	1.0832	1.2395	1.1856
1.178	1.1673	1.3495	1.2467	1.0791	1.4686	0.9005	1.2548	1.0991
0.8619	0.9317	1.0295	0.8664	0.9619	0.9804	1.038	0.8897	0.8492
0.7515	0.774	0.8937	0.6494	0.7319	1.1103	0.933	0.7012	0.729
0.9041	0.8526	0.869	0.9018	0.8457	0.6676	0.8894	0.8486	0.8373
1.1011	0.9917	0.9309	0.6796	0.7136	0.7099	1.1449	1.0848	0.7879
1.096	0.9781	1.0141	0.9489	0.8796	0.6853	1.485	1.1227	0.9448
0.8699	0.8643	1.003	0.8842	0.9553	0.9229	0.8044	0.9433	0.9744
0.9829	0.9046	0.6795	0.8511	0.8324	0.5604	0.726	0.8615	0.8901
0.6308	0.7054	0.626	0.6819	0.5729	0.5516	0.7461	0.7707	0.6886
1.2841	1.0677	1.2356	1.2012	0.9123	0.686	1.0194	1.2181	0.9259
0.9551	0.965	0.8829	0.5732	0.7197	0.8436	0.8564	1.0265	0.7807

0.9695	0.9003	0.8202	0.5041	0.692	0.9384	0.9921	0.9306	0.7519
0.9239	0.9011	0.989	0.8867	0.8632	0.8005	0.9829	0.9495	0.9318
1.2707	1.235	1.0886	1.244	1.3008	1.533	1.112	1.1344	1.2164
0.8531	0.9983	0.9754	1.0045	1.1439	1.0098	0.8096	0.9447	1.2133
0.6615	0.9008	0.998	0.6744	0.999	0.7665	1.1183	0.8898	1.0057

A. thaliana-re	A. thaliana-u	C. variabilis-u	O. sativa-revi	O. sativa-unr	O. tauri-unre	P. patens-unr	P. trichocarpa	Z. mays-unreview
1.044	1.1684	1.0889	0.9404	0.9681	0.8773	1.0518	1.1606	1.206
0.9132	1.1399	1.0377	1.505	1.3538	1.4339	1.143	0.941	1.2299
0.7591	0.9875	0.9583	1.2423	1.2117	0.8112	1.1106	0.8945	1.0577
1.4423	1.2768	1.2076	1.3412	1.212	1.2763	1.0143	1.3189	1.233
1.1137	0.9905	1.166	1.0606	1.0219	1.2405	1.1662	1.0927	1.0238
0.9345	1.0274	0.9842	0.9246	0.9943	0.9252	1.1019	0.9632	0.9362
0.8808	0.8094	0.8937	0.7885	0.7925	1.0345	0.7825	0.7626	0.7791
0.9906	0.8835	0.8218	1.1056	0.9002	0.6546	0.8416	0.8888	0.8184
1.0864	0.8853	0.7447	0.7114	0.6659	0.7211	0.7964	0.9684	0.7414
1.0129	0.9459	0.9498	0.9555	0.859	0.7556	1.0463	1.0658	0.8788
0.8377	0.888	0.9528	1.0668	0.8601	0.869	0.9278	1.0141	1.037
0.7386	0.8421	0.4605	0.7606	0.7833	0.523	0.8003	0.75	0.8348
0.6552	0.706	0.6987	0.3964	0.5565	0.5565	0.6758	0.8099	0.6186
1.0801	1.0356	1.3177	0.7402	0.8568	0.6977	1.0189	1.1111	0.9061
1.0137	0.9696	0.8338	0.5395	0.7155	0.6064	0.8041	1.0478	0.7542
1.0022	0.8928	0.9238	0.5512	0.6923	0.8293	0.9548	0.9073	0.7771
0.9959	0.9517	1.0302	0.9957	0.9315	0.8204	0.8681	1.0201	0.9799
1.1582	1.1815	1.1406	1.2478	1.2644	1.638	1.127	1.082	1.235
0.8653	1.0174	1.0818	0.87	1.149	1.1525	1.4259	0.9602	1.2161
0.718	0.9342	0.7875	0.7277	0.9517	0.7282	1.0132	0.8898	0.8722

Supplementary figure 1. All six major human NAT complexes (NatA-F) were most likely present in the Last Eukaryotic Common Ancestor (LECA).

Catalytic and regulatory subunits of all six major human NATs complexes were identified across the eukaryotic tree of life, suggesting they were all present in the LECA. NATs subunit orthologs were identified in 73 species representative of the eukaryotic tree of life[59-63]. Results are indicated according to reciprocal blastp E-value score ("filled dot"= E-value score lower than e^{-8}; "open dot"= E-value score between e^{-8}-e^{-03}; "no dot"= E-value score more than e^{-03}. Known subunits of *H. sapiens* NATs were used as reference for bidirectional blastp, except in the case of fungi where *S. cerevisiae* NATs were used instead[9, 83]. Black dot indicates NAT was identified using *H. sapiens* ortholog; orange dot indicates that NAT was identified using *S. cerevisiae* ortholog; green, red, yellow and blue dots indicate that NATs were identified, respectively, using the phylogenetically closest plant, chromalveolata, excavate and microsporidia species ortholog. In the case of species-specific gene duplication, the number of dots is equivalent to the number of identified NAT paralogs. Phylogenetic distribution shown in this figure was previously reported[59-63].

Supplementary figure 2. Identified orthologs of Naa10 and Naa50 are most likely catalytically active.

Three major catalytically active residues in Naa10 (α1–α2 loop 'E'; β5 helix 'R'; β6-7 helix 'Y') and two in Naa50 (β4 helix 'Y'; β5 helix 'H') were recently described[64, 65]. Presence of the key catalytically active residues of Naa10 (E, R, Y; Glutamic acid, Arginine, and Tyrosine) and Naa50 (Y, H; Tyrosine and Histidine) is indicated for 27 species representative of the eukaryotic tree of life. Substitution of these canonical catalytically active residues is displayed by showing a distinct amino acid at the respective position. Phylogenetic distribution shown in this figure was previously reported[59-63].

Supplementary figure 3. Identified orthologs of Naa10 and Naa50 in fungi, microsporidia, and excavata are most likely catalytically active.

Three major catalytically active residues in Naa10 (α1–α2 loop 'E'; β5 helix 'R'; β6-7 helix 'Y') and two in Naa50 (β4 helix 'Y'; β5 helix 'H') were recently described[64, 65]. Presence of the key catalytically active residues of Naa10 (E, R, Y; Glutamic acid, Arginine, and Tyrosine) and Naa50 (Y, H; Tyrosine and Histidine) is indicated for 29 species (thirteen fungi, ten microsporidia, six excavata). Substitution of these canonical catalytically active residues is displayed by showing a distinct amino acid at the respective position. Phylogenetic distribution shown in this figure was previously reported[59-63].

Supplementary figure 4. Orthologs of Naa10 were not identified in several species of birds.

Orthologs of Naa10 (the catalytic subunit of NatA) were not identified in *Gallus gallus* (chicken), *Meleagris gallopavo* (turkey), *Taenopygia guttata*, *Ficedula albicollis*, and *Melopsittacus undulatus* (budgerigar). This absence was further confirmed by HMMER (data not shown). Naa10 orthologs were nevertheless identified in *Falco cherrug* (falcon) and *Anas platyrhynchos* (duck). Orthologs for Naa15 (regulatory subunit of NatA) and all other NATs subunits were identified in seven avian species representative of the major clades of birds phylogenetic tree[91]. Results are indicated according to reciprocal blastp E-value score ("filled dot"= E-value score lower than e^{-8}; "open dot"= E-value score between e^{-8}-e^{-03}; "no dot"= E-value score higher than e^{-03}. In the case of species-specific gene duplication events, the number of dots is equivalent to the number of identified paralogs. Phylogenetic distribution shown in this figure was previously reported[60, 63, 91].

Supplementary figure 5. Lack of detectable residue usage frequency bias for the N-terminal third, fourth, fifth and sixth position.

Fold enrichment heat maps of each amino acid usage frequency at the N-terminal third **(A)**, fourth **(B)**, fifth **(C)**, and sixth **(D)** positions when compared to the total proteome. When compared to the N-terminal second position (Fig.

5), the N-terminal third, fourth, fifth, and sixth residues positions show significantly less amino acid usage frequency biases across the eukaryotic tree of life. Amino acid usage frequency bias for each N-terminal position was analyzed by calculating the amino acid usage frequency for each position divided by its frequency in the total proteome (for more experimental detail see material and methods). The fold enrichment heat map shows the over-representation range (>1) of each amino acid at the N-terminal third/fourth/fifth/sixth positions when compared to the total proteome. For each species and for each amino acid, it was attributed a black color (≤1.0) when the amino acid is under-represented in the N-terminal third/fourth/fifth/sixth positions compared to its total proteome usage frequency. A detailed breakdown of the values used in this heat map is shown in Supplementary Table 2.

Supplementary table 1. Protein accession numbers and reciprocal blastp E-value scores for all identified NATs.

Supplementary table 2. Fold enrichment values of each amino acid at the N-terminal second, third, fourth, fifth, and sixth positions when compared to total proteome usage.
